# Fragmentation Pattern-Based Screening Strategy Combining Diagnostic Ion and Neutral Loss Uncovered Novel *para*-Phenylenediamine Quinone Contaminants in the Environment

**DOI:** 10.1021/acs.est.4c00027

**Published:** 2024-03-21

**Authors:** Wei Wang, Guodong Cao, Jing Zhang, Weixia Chang, Yuecheng Sang, Zongwei Cai

**Affiliations:** State Key Laboratory of Environmental and Biological Analysis, Department of Chemistry, Hong Kong Baptist University, Hong Kong SAR 999077, China

**Keywords:** para-phenylenediamine quinones, 6PPD-Q, high-resolution mass spectrometry, emerging contaminants, nontargeted identification, neutral loss, diagnostic fragment

## Abstract

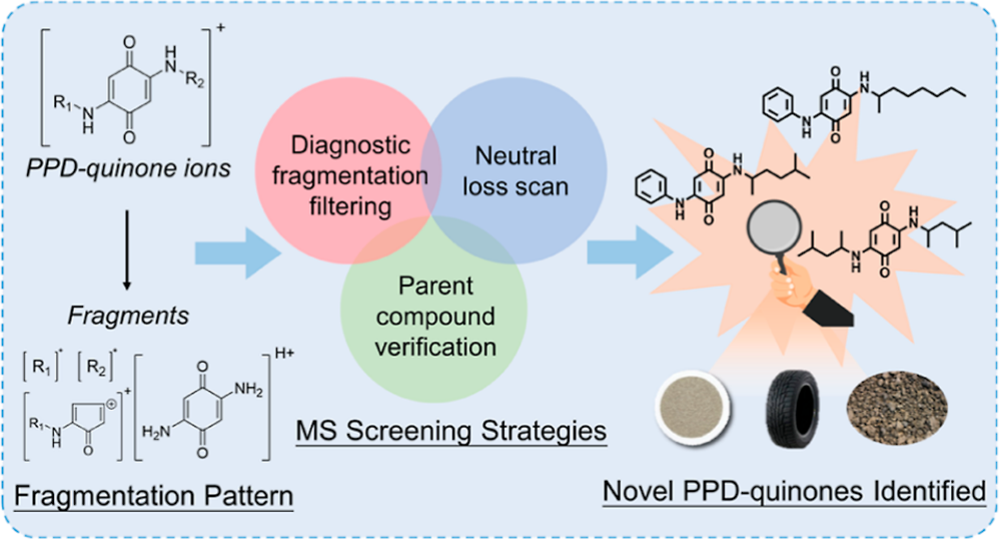

Identifying transformed emerging contaminants in complex environmental compartments is a challenging but meaningful task. Substituted *para*-phenylenediamine quinones (PPD-quinones) are emerging contaminants originating from rubber antioxidants and have been proven to be toxic to the aquatic species, especially salmonids. The emergence of multiple PPD-quinones in various environmental matrices and evidence of their specific hazards underscore the need to understand their environmental occurrences. Here, we introduce a fragmentation pattern-based nontargeted screening strategy combining full MS/All ion fragmentation/neutral loss-ddMS^2^ scans to identify potential unknown PPD-quinones in different environmental matrices. Using diagnostic fragments of *m*/*z* 170.0600, 139.0502, and characteristic neutral losses of 199.0633, 138.0429 Da, six known and three novel PPD-quinones were recognized in air particulates, surface soil, and tire tissue. Their specific structures were confirmed, and their environmental concentration and composition profiles were clarified with self-synthesized standards. *N*-(1-methylheptyl)-*N*′-phenyl-1,4-benzenediamine quinone (8PPD-Q) and *N*,*N*′-di(1,3-dimethylbutyl)-*p*-phenylenediamine quinone (66PD-Q) were identified and quantified for the first time, with their median concentrations found to be 0.02–0.21 μg·g^–1^ in tire tissue, 0.40–2.76 pg·m^–3^ in air particles, and 0.23–1.02 ng·g^–1^ in surface soil. This work provides new evidence for the presence of unknown PPD-quinones in the environment, showcasing a potential strategy for screening emerging transformed contaminants in the environment.

## Abstract 2

Identifying emerging contaminants in complex environmental compartments is challenging. This work established a fragmentation pattern-based mass spectrometry method and uncovered the widespread occurrence of three novel PPD-quinones in the environment.

## Introduction

Substituted *para*-phenylenediamine quinones (PPD-quinones) are a class of emerging contaminants that originate from the oxidation of tire rubber antioxidant PPDs. One of them, *N*′-phenyl-*p*-phenylenediamine quinone (6PPD-Q), was identified to be the culprit behind the urban runoff mortality syndrome that can lead to the acute death of coho salmon (*Oncorhynchus kisutch*) at a trace level (24 h-LC_50_ 95 ng/L).^1,2^ The contaminant was proven to be acutely fatal to rainbow trout (*Oncorhynchus mykiss*), brook trout (*Salvelinus fontinalis*),^3^ white-spotted char (*Salvelinus leucomaenispluvius*),^4^ and toxic to zebrafish,^5^*Gobiocypris rarus*, and other aquatic species.^6^ Besides aquatic species, recent evidence suggests that 6PPD-Q can also cause side effects in terrestrial organisms, including intestinal toxicity, abnormal locomotion, neurodegeneration, and reduced reproductive capacity in *Caenorhabditis elegans*,^7−9^ as well as hepatotoxicity and multiple organ injury in mice.^10,11^ These noxious effects have drawn significant concern about 6PPD-Q and its analogues, especially those unidentified emerging quinone contaminants. Our early study uncovered the occurrence of a range of emerging PPD-quinones, such as *N*-isopropyl-*N*′-phenyl-1,4-phenylenediamine quinone (IPPD-Q), *N*,*N*′-bis(methylphenyl)-1,4-benzenediamine quinone (DTPD-Q), *N*-phenyl-*N*′-cyclohexyl-*p*-phenylenediamine quinone (CPPD-Q), and *N*,*N*′-diphenyl-*p*-phenylenediamine quinone (DPPD-Q), in ambient environments of water, air, and soil.^12^ By applying a suspect screening strategy, another new PPD-quinone, *N*,*N*′-bis(1,4-dimethylpentyl)-*p*-phenylenediamine quinone (77PD-Q), was also frequently identified in the air particulates collected in China.^13^ These PPD-quinone contaminants were not only pervasively distributed in various environmental compartments such as rubber products,^14^ dust,^15^ electronic waste,^16^ runoff water,^17^ sediments,^18^ and urban water systems,^19^ but also detectable in human urine.^20^ Additionally, these quinone contaminants were found to possess oxidative potentials,^21^ and induce bioluminescence inhibition to aquatic bacterium.^22^ These discoveries have revealed the prevalence of PPD-quinone as an emerging class of contaminants and confirmed their inevitable exposure to humans. Given the serious ecological hazards and potential health risks as well as the deficiency in the identification of emerging PPD-quinones, it is imperative to build comprehensive profiles by screening unknown PPD-quinones in the environment.

As PPD-quinones are undocumented transformation products (TPs) that are derived from the natural oxidation of PPDs, targeted identification with commercial standards is inaccessible. Nontargeted identification using high-resolution mass spectrometry (HRMS) enables the recognition of novel compounds without standards and is thus considered as a powerful tool for identifying novel environmental contaminants hitherto unattainable.^23,24^ It possesses the capability to analyze complex samples, providing high-confidence identification of thousands of specific molecular components from a single analysis.^25,26^ By coupling MS with separation means including gas/liquid chromatography (GC/LC) system,^1,23^ applying nontargeted MS acquisitions such as full scan, data-dependent acquisition, and data-independent acquisition,^27,28^ and leveraging multiple screening strategies based on the fragment and/or isotopic pattern,^29,30^ a great range of emerging contaminants, accompanied by their congeners have been identified.^31−33^ Recently, appreciable efforts have been made to leverage HRMS for screening PPD-quinones in various environments. Tian et al. first isolated 6PPD-Q in tire wear particle leachate using multidimensional chromatography, inferred its parent compound, and accordingly confirmed its structure using GC/UPLC-HRMS and nuclear magnetic resonance (NMR) analyses.^1^ Using an ozone-synthesized standard, they quantified the levels of 6PPD-Q in urban roadway runoff and receiving waters. Later, Cao et al. confirmed the occurrence of 6PPD-Q in runoff water, roadside soil, and air particulates with a parallel comparison of the MS^2^ spectrum determined by Tian et al.^12^ Using an HRMS-based suspecting screening strategy, they identified four other PPD-quinones and measured their concentrations in various environmental matrices with self-synthesized standards. Through adopting a strategy of suspect screening, MS^2^ identification, and self-synthesized standard confirmation, Wang et al. have discovered another PPD-quinone of 77PD-Q, which was prevalently detected in the airborne particulates collected in China.^13^ Most recently, Zhao et al. conducted suspect screening for PPD-quinones in crumb rubber and elastomeric consumer products and found the occurrence of *N*-(1,4-dimethylpentyl)-*N*′-phenylbenzene-1,4-diamine quinone (7PPD-Q) in tire wear particle and crumb rubber.^14^ These works confirmed the validity of HRMS in recognizing novel PPD-quinones and implied a wide range of such contaminants in the surrounding environment. However, the suspect screening strategies need to have basic information (e.g., exact mass) about the suspects and are often limited by high false-positive rates and high uncertainties.^34^ Investigations focusing on the comprehensive screening of unknown PPD-quinones among multiple environmental matrices with nontargeted approaches are still scarce. For systematically revealing the occurrence and compositional characteristics of potential unknown PPD-quinones, the development of a rapid and efficient screening approach for their specific identification is crucial.

In this study, we devised a fragmentation pattern-based MS approach aimed at comprehensively investigating the known and potential unknown PPD-quinone contaminants in the environment. A mass fragmentation pattern model was first established based on the characteristic fragment ions and neutral losses of known PPD-quinones. Featured product ions and neutral losses with high specificity were selected as markers for screening novel PPD-quinones. Various environmental samples, including tire tissue, atmospheric particulate matter, and surface soil, were analyzed through the multistage mass spectrometric analysis combining full MS, all ion fragmentation (AIF), and neutral loss (NL) ddMS^2^ scans. Two novel PPD-quinones were identified for the first time, and their environmental concentrations were accurately quantified using self-synthesized standards.

## Materials and Methods

### Chemicals and Standards

Chemicals used for synthesis, including 2-aminooctane, 2-amino-5-methylhexane, benzoquinone, and 4-methylpentan-2-amine, were purchased from TCI (Japan) with purities over 98%, while 2-anilino-1,4-benzoquinone was prepared as described elsewhere.^12^ PPD-quinone standards, including IPPD-Q, DPPD-Q, CPPD-Q, DTPD-Q, 6PPD-Q, 77PD-Q, and the internal standard of deuterated 6PPD-Q (6PPD-Q-*d*_5_), were synthesized in our laboratory with purities higher than 95%.^12,13^ Authentic standards of their parent PPDs were obtained from J&K Chemical Company and AccuStandard (Hong Kong). Surrogate standard diphenylamine-*d*_10_ was obtained from the TRC (Burlington, Canada). Newly identified 7PPD-Q, 8PPD-Q, and 66PD-Q were synthesized in our laboratory and characterized using NMR (^1^H and ^13^C, Bruker Avance-III 400 MHz, USA), HRMS (Q Exactive, Thermo Fisher Scientific, USA), and infrared spectroscopy (Spectrum Two FT-IR, PerkinElmer, USA). The detailed synthesis schemes and measurements are described in Text S1. HPLC-grade solvents, including dichloromethane and acetonitrile purchased from VMR Chemicals (Hong Kong), were adopted in this study.

### Sample Collection

Samples were collected from multiple environmental compartments, including air particulates (fine particulate matter with an aerodynamic diameter less than 2.5 μm, PM_2.5_), tire tissue, and surface soil. Among these samples, air particulate samples (*n* = 18) were collected in Taiyuan, China, using quartz fiber filters (QMA, 90 mm, Whatman International Ltd., UK) employing a medium-volume air sampler (AMAE Co. Ltd., Shenzhen, China) throughout January to December 2018. The quartz fibers were preheated at 550 °C to eliminate possible contamination, and a 24-h composite sample was collected. After collection, the filters were wrapped in aluminum foil and stored in a −80 °C freezer for further pretreatment. Tire tissue samples (*n* = 8) were collected from tires with usage time of zero (new) to four years from an auto repair shop. Each sample was collected from six different sites on a tire (inner and outer layers), crushed using a shredder, and then mixed into an integrated sample. Surface soil samples (*n* = 20) were collected from green belt areas on the side of four main roads of Hong Kong on nonrainy days in August and September 2021. At least three replicate soils from each site were collected and then blended into a composite sample. All the samples were transferred to the laboratory within 2 h and immediately weighed for 10–30 g. After freeze-drying and homogenization, the samples were sieved through a 60-mesh screen and stored in a −20 °C freezer for further treatments.

### Sample Extraction and Pretreatment

The pretreatment procedures for each type of sample followed the methods outlined in our previous studies.^12,13^ The filters absorbing air particulates were shredded and spiked with surrogate standards of 20 ng. Subsequently, ultrasonic extraction was performed twice (each for a duration of 15 min) using 5 mL of dichloromethane per extraction, followed by an additional 15 min ultrasonication with 5 mL of acetonitrile. The extracts were merged and evaporated under nitrogen to near dryness. Following the solvent exchange with 1 mL of acetonitrile, the extract was filtered using a 0.2 μm PTFE organic membrane filter. Before instrument analysis, 20 ng of the internal standard was spiked. The extraction of tire tissue and surface soil follows similar procedures. Generally, 100 mg of each pretreated tire tissue and surface soil sample was spiked with 20 ng of the surrogate standard. Each sample was extracted with dichloromethane (2 mL × 2) and acetonitrile (2 mL × 1) for 15 min each. The extracts were purged with nitrogen to near dryness and redissolved in acetonitrile. The samples were filtered through a 0.2-μm syringe filter and then spiked with 20 ng of internal standard before further analysis.

### Instrument Analysis

Ultraperformance liquid chromatography (Vanquish MD) integrated with electrospray ionization (ESI) Q Exactive hybrid quadrupole-Orbitrap mass spectrometry (UPLC-ESI-Q Orbitrap MS, Thermo Fisher Scientific, USA) was used to achieve the screening of PPD-quinones in the modes of Full MS/AIF/NL ddMS^2^ and parallel reaction monitoring (PRM). The MS system was precalibrated, using calibration solutions according to the manufacturer’s guidelines (Thermo Fisher Scientific, USA), to ensure the mass accuracy of the measurements within 5 ppm. The quantification was performed using the same HPLC system coupled with an ESI TSQ Altis triple quadrupole mass spectrometer (HPLC-ESI-QqQ MS, Thermo Fisher Scientific, USA) in multiple reaction monitoring (MRM) mode. Chromatographic separation was carried out using an Acquity HSS T3 column (1.8 μm, 2.1 × 100 mm) with mobile phases composed of 0.1% (v/v) formic acid in Milli-Q water (A) and acetonitrile (B) at a flow rate of 0.3 mL·min^–1^. The elution protocol consisted of an initial 1 min period with 2% B, followed by a linear increase to 100% B over 19 min, held for 3 min, then a return to 2% B over 1 min, and a subsequent equilibration for 3 min. All analytes were quantified using their respective calibration curves, except for 66PD, which was semiquantified using the calibration curve of 77PD due to the unavailability of a specific reference standard. Detailed instrumental parameters, including resolutions, gas flows, voltages, scan ranges, temperatures used in HRMS, as well as quantify/qualify ion pairs and optimized collision energies used in QqQ MS, are listed in Tables S1 and S2.

### Quality Assurance and Control

To guarantee high-quality data, we implemented strict quality assurance and control procedures. To assess potential contaminants from the sampling and pretreatment processes, field and procedure blank samples using a blank filter or blank container devoid of tire tissue/soil were adopted. These blanks were analyzed by using the same method as the samples. Among the analytes, it was noted that CPPD-Q, DPPD-Q, and IPPD-Q exhibited detectable levels in the procedural blank, albeit at concentrations below 2% of their respective quantified levels. These detected levels were accounted for and subtracted from the background levels. For evaluating method recoveries, a total of six replicates of PM_2.5_ loaded filters, tire tissues, and surface soils were fortified with 10 ng of the target analytes individually. Recovery calculation was performed using detected concentrations in spiked samples, corrected for detection in unspiked samples over nominal spike concentrations. The recoveries of these target analytes obtained from the spiking analysis ranged from 67 ± 3 to 94 ± 14% for air particulates, 72 ± 12 to 117 ± 20% for tire tissues, and 64 ± 14 to 105 ± 18% for surface soils (Table S2). The method’s repeatability was assessed through duplicate testing for every set of eight samples, yielding standard deviations below 20% in all cases. The calibration curves for each analyte were generated by using acetonitrile as the solvent, demonstrating regression coefficients exceeding 0.99. In instances in which sample concentrations exceeded the range of the calibration curve, appropriate dilutions were performed. The determination of the limit of detection (LOD) and limit of quantitation (LOQ) for different analytes depended on their presence in blank samples and their recovery rates (Table S2). For analytes detected in blank samples, LOD/LOQ values were defined as 3/10 the standard deviation of the procedural blank. For analytes not detectable in blank samples, LOD/LOQ values were calculated as 3 times/10 times the signal-to-noise ratios of the lowest detectable levels for the standards dissolved in the matrix.

### Method Development and Data Processing

**Figure 1:**
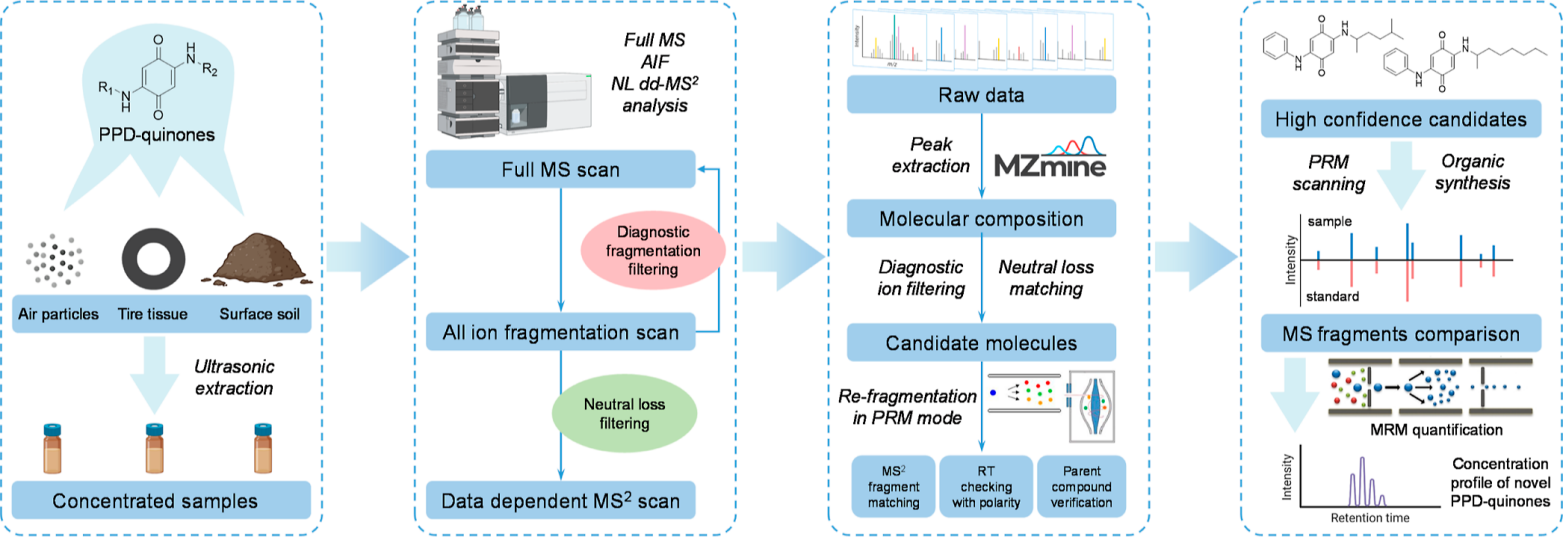
Workflow of diagnostic fragmentation filtering coupled with neutral loss scan strategy for the screening of PPD-quinones.

**Figure 2:**
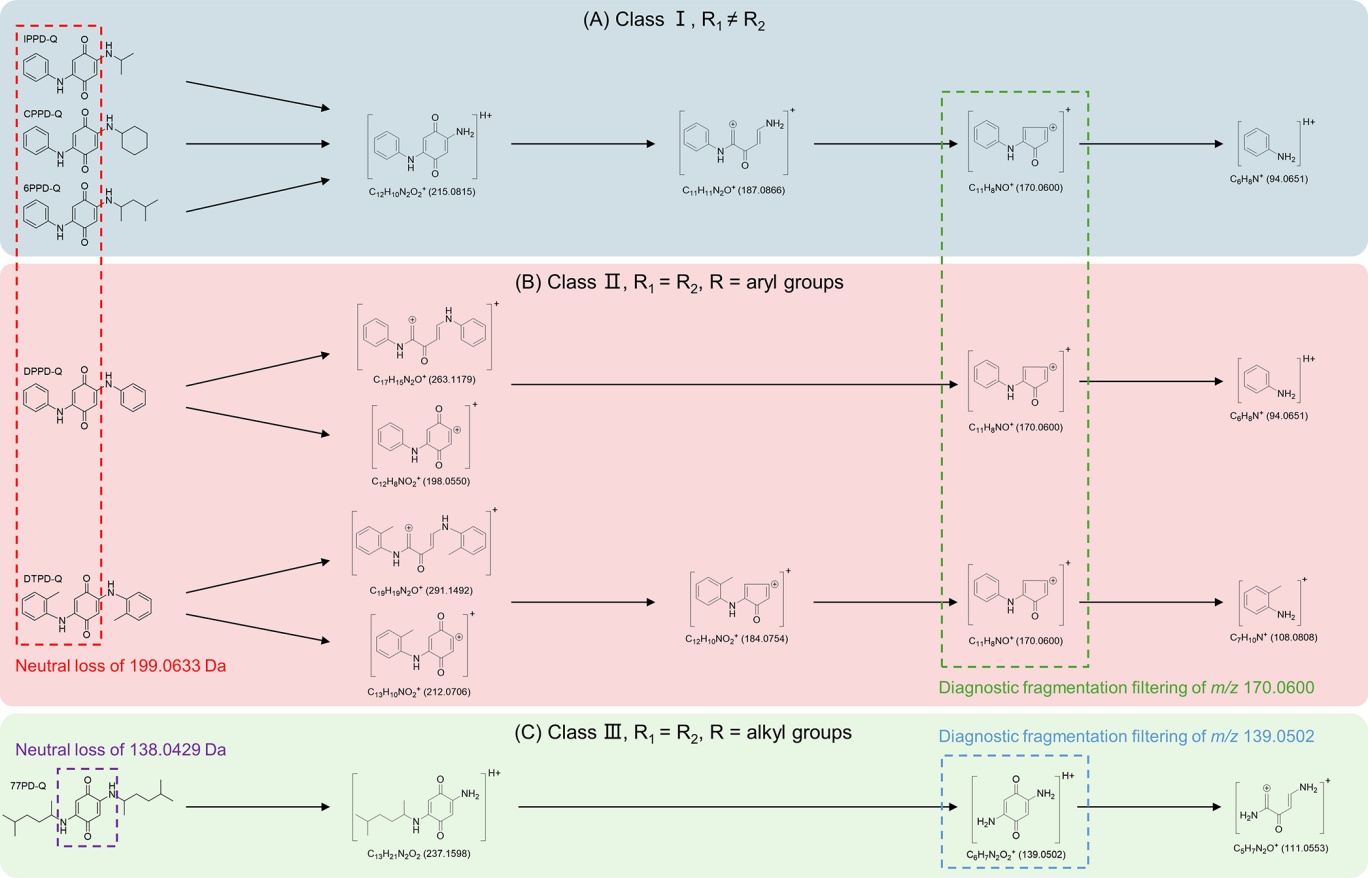
Fragmentation pattern of PPD-quinones (IPPD-Q, CPPD-Q, 6PPD-Q, DPPD-Q, DTPD-Q, and 77PD-Q) obtained from positive parallel reaction monitoring mode.

To address the challenges of high-efficiency screening for unknown PPD-quinone contaminants in the environment, a systematic workflow utilizing a nontargeted analysis (NTA) approach is illustrated in Figure 1. After extraction, the samples were analyzed via UPLC-ESI-Q Orbitrap MS in full MS/AIF/NL dd-MS^2^ mode. In this mode, a full scan was performed first to obtain the total MS^1^ spectra, followed by an AIF scan to acquire the full MS^2^ spectra. In addition to this “global” scanning, acquisition of specific molecules was also triggered to obtain their MS^2^ spectra if a component loses a neutral fragment matching the set NL filters. This scanning mode enabled the screening of specific molecules using two strategies: first, the AIF spectra can filter molecules with diagnostic fragmentations; second, data-dependent MS^2^ via NL filters can identify molecules with specific fragmentation behaviors. Consequently, this method can serve as a high-throughput and efficient assay for identifying novel contaminants with specific functional groups.^35−37^ The obtained data were then processed using MZmine with peak extraction and diagnostic fragmentation filtering (DFF) modules.^38^ For the detection of PPD-quinones having different substitutions or same aryl substitutions in the side chains (Classes I and II in Figure 2), the DFF module conducted a search for MS^2^ spectra containing product ions with an *m*/*z* value of 170.0600 with a normalized peak intensity at least 10% of the base peak. For the detection of PPD-quinones having the same alkyl substitutions in the side chains (Class III in Figure 2), the DFF module conducted search for MS^2^ spectra featuring product ions with an *m*/*z* of 139.0502. Voltage applied to the ESI source may lead to the generation of premature ion fragments, commonly referred to as in-source fragments.^39^ To mitigate this issue, a thorough manual confirmation was conducted on all potential matches to verify their identity as molecular ions rather than as in-source fragments. Molecules that fit the DFF criteria were further compared with those obtained from NL channels to achieve a highly specific identification. The candidate molecules, with their retention times (RTs) determined, were fragmented in PRM mode using varying collision energies: (1) to cross-comparison MS^2^ fragments with those of known PPD-quinones for structural information; (2) to acquire structure-related polarity information by comparing their RTs; (3) to confirm their identity by checking their neutral losses and diagnostic fragments. For identified PPD-quinones, the presence of their parent compounds was also suspect-screened and confirmed with standards, as most of the PPD-quinone compounds are considered to have transformed from their parent PPDs.^1,12,13^ The NTA Study Reporting Tool (SRT) was adopted for the evaluation of the nontargeted screening analyses.^40^ The CH_2_–based Kendrick mass defect (KMD) of each molecule was obtained in reference to early works.^41,42^ Its detailed calculation and more statistical analysis are described in the Supporting Information.

## Result and Discussion

### Fragmentation Pattern Recognition of PPD-Quinones

To better determine these emerging contaminants in multiple environmental matrices, it is of great significance to develop a method of identification with high specificity. Characterizing their MS fragmentation behaviors, thus summarizing the fragmentation pattern, is fundamental and essential. PPD-quinone compounds have a central *p*-phenylenediamine quinone structure, with two amino groups located at the para position connected with two side chain substitutions R_1_ and R_2_ (Figure 1). Here, we assessed the fragmentation pathways of six known PPD-quinones to obtain their MS fragmentation rules. The MS^2^ spectra of each PPD-quinone under varying normalized collision energies (NCE) of 10–60% are summarized in Figures S1–S6. According to the structure of their substituted groups (R_1_ and R_2_), these PPD-quinones have been categorized into three classes, including Class I, R_1_ ≠ R_2_; Class II, R_1_ = R_2_, R = aryl groups; Class III, R_1_ = R_2_, R = alkyl groups. For Class I containing PPD-quinones that have different substituted groups, that is, IPPD-Q, CPPD-Q, and 6PPD-Q, their characteristic fragmentation rules are shown in Figure 2A. It can be observed that they share the same fragment of *m*/*z* 215.0815 by losing their respective R_2_ substituted groups, that is, isopropyl for IPPD-Q, cyclohexyl for CPPD-Q, 4-methylpentan-2-yl for 6PPD-Q. By further loss of a carbonyl group (C=O, 27.9949 Da), a characteristic product ion (*m*/*z* 187.0866) was formed. This ion can undergo further fragmentation by losing an amino group (NH_3_, 17.0265 Da) and forming a more stable five-membered ring with the characteristic ion C_11_H_8_NO^+^ (*m*/*z* 170.0600). In addition, the fragment ion at *m*/*z* 94.0651 is ascribed to the presence of an aniline ion (C_6_H_8_N^+^). Upon summarizing these fragments of PPD-quinones in Class I, we can observe high consistency in their fragmentation behaviors, characterized by the gradual loss of (1) saturated aliphatic substituents, (2) a carbonyl group, (3) an amino group, and (4) a cyclopentadienone. For Class II containing PPD-quinones that have the same substituted aryl groups, that is, DPPD-Q, DTPD-Q, their fragmentation rules are shown in Figure 2B. It is noted that besides the fragmentation in the side chain substitutes (R_2_), an apparent cleavage of the quinone moiety with loss of the carbonyl group can be found. This may be due to the relatively stable side-chain aromatic structure, thus making the carbonyl groups of the central quinone the initial site of the fragmentation. A further fragmentation in the center benzoquinone carbonyl group was observed for DPPD-Q and DTPD-Q, yielding product ions of *m*/*z* 170.0600 and *m*/*z* 184.0757. The product ion of *m*/*z* 184.0757 can be further fragmented, with the loss of a methylene (CH_2,_ 14.0157 Da), to form the product ion of *m*/*z* 170.0600 in line with other PPD-quinones. It is worth noting that the fragmentation of DPPD-Q and DTPD-Q demonstrated high consistency with molecules in Class I that underwent a neutral fragment loss of C_5_H_4_O (80.0262 Da), yielding fragments of the aniline ion (*m*/*z* 94.0651) and the *o*-toluidine ion (*m*/*z* 108.0808). For Class III containing PPD-quinone that has the same substituted alkyl groups, that is, 77PD-Q, its fragmentation pattern followed the rules shown in Figure 2C. The fragmentation pathway of 77PD-Q involves the gradual loss of two substituted 2-methylhexyl (C_7_H_16_, 100.1252 Da), yielding a product ion of C_6_H_7_N_2_O_2_^+^ (*m*/*z* 139.0502). This fragment could undergo further fragmentation with a loss of a carbonyl group, thus forming a product ion of C_5_H_7_N_2_O^+^ (*m*/*z* 111.0553). It should be noted that the current reported PPD-quinone that fits such a structural pattern contains only 77PD-Q. Further investigation to identify potential contaminants of this class is necessary for elucidating and verifying their MS fragmentation patterns.

By summarizing the fragmentation patterns of PPD-quinones described above, we observed a consistent neutral loss of the carbonyl group, which was attributed to the cleavage of the central benzoquinone across all PPD-quinone contaminants under various collision energy conditions. Furthermore, the neutral structure of C_12_H_9_NO_2_ (199.0633 Da, red dashed line in Figure 2) for PPD-quinones in Class I and II, and C_6_H_6_N_2_O_2_ (138.0429 Da, purple dashed line in Figure 2) for Class III, could also be considered as potential screening filters. These structures possess the most distinctive features of PPD-quinones, which are crucial for achieving a high specificity in their recognition. Additionally, the same product fragment C_11_H_8_NO^+^ (*m*/*z* 170.0600, green dashed line in Figure 2) possessed by different PPD-quinones makes diagnostic fragmentation filtering a possible approach to their recognition. For potential PPD-quinones that may be classified in Class III, the product ion of C_6_H_7_N_2_O_2_ (*m*/*z* 139.0502, blue dashed line in Figure 2) can be selected as a tracking fragment.

### Method Validation with Known PPD-Quinones

**Figure 3:**
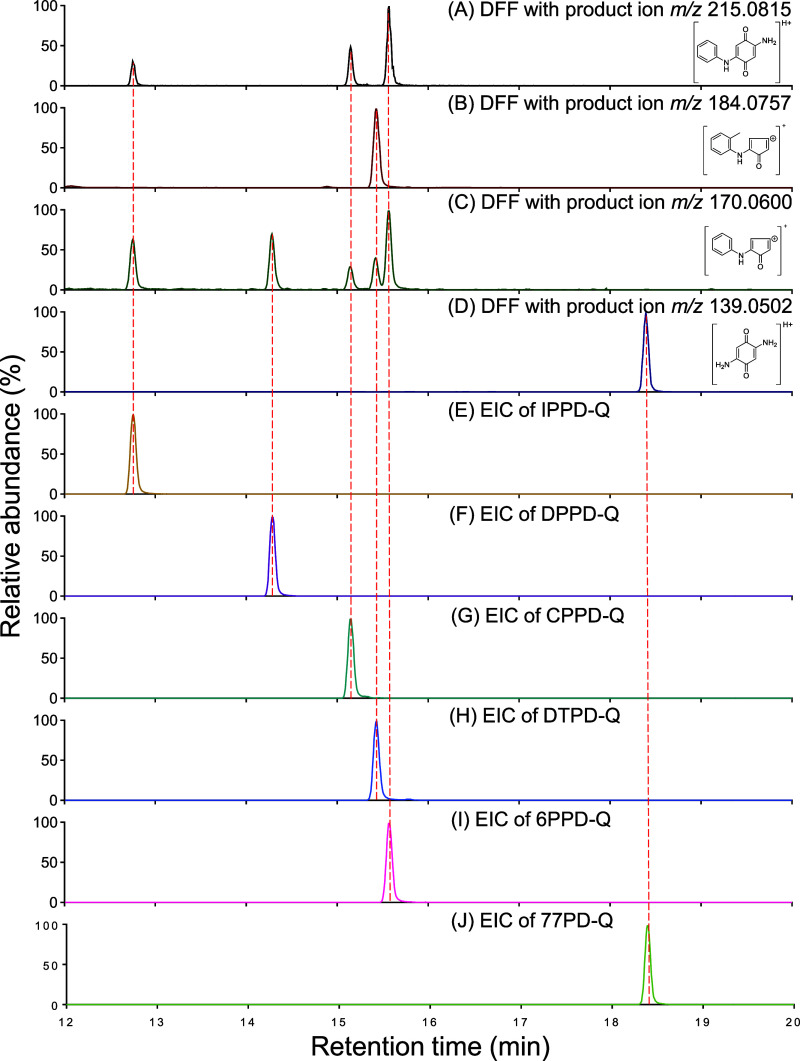
Method validation with the recognition of six PPD-quinones. Diagnostic fragmentation filtering with different product ions (A–D) can achieve the recognition of various PPD-quinones (E–J). Among them, product ion of C_11_H_8_NO^+^ (*m*/*z* 170.0600, C) can be adopted to identify IPPD-Q (E), DPPD-Q (F), CPPD-Q (G), DTPD-Q (H), and 6PPD-Q (I), while product ion of C_6_H_7_N_2_O_2_^+^ (*m*/*z* 139.0505, D) can be applied to identify 77PD-Q (J).

To verify the feasibility of the method, a mixed standard sample containing all six known PPD-quinones was adopted for “blind screening.” First of all, the characteristic product ions of PPD-quinones demonstrated in Figure 2 were adopted as diagnostic ions to screen the candidates. Among these ions, it is observed that *m*/*z* 215.0815, 184.0757, and 170.0600 demonstrated typical identification features. As Figure 3 shows, monitoring the fragment ion of *m*/*z* 215.0815 led to the identification of PPD-quinones with R_1_ ≠ R_2_ (IPPD-Q, CPPD-Q, and 6PPD-Q). Comparatively, *m*/*z* 184.0757 can be utilized specifically to track DTPD-Q as its characteristic product ion. It is noteworthy that product ion *m*/*z* 170.0600 enables efficient recognition of five PPD-quinone contaminants from Class I and II and thus can be considered a “universal” marker ion for their identification. It is reasonable as all the PPD-quinones could produce such fragment ions with different collision energies (Figures S1–S6). For PPD-quinones in Class III, the diagnostic ion of *m*/*z* 139.0502 was found to have a perfect match of RTs with the molecular ion of 77PD-Q, demonstrating its ability to track alkyl-substituted symmetric quinones. In addition, we optimized the collision energy of the AIF mode to achieve the best recognition effects. The result indicates that with an NCE value of 50%, the intensity of the identified PPD-quinones is generally the highest, with each component demonstrating clear chromatography peaks and less background noise (Figure S7).

In parallel, we have also adopted the neutral loss mode as a screening strategy to identify these contaminants. Multiple neutral fragments were tested, including CO (27.9949 Da), C_5_H_4_O (80.0262 Da), C_12_H_9_NO_2_ (199.0633 Da), and C_6_H_6_N_2_O_2_ (138.0429 Da). The latter two were selected as primary filters to detect PPD-quinone suspects, because the results indicated that all six PPD-quinones could be successfully recognized in the obtained MS transitions. These transitions showed a perfect match of EIC peaks corresponding to their molecular ions and neutral losses (Figure S8). All of the standard compounds were screened using two strategies that took into account both their NL behaviors and diagnostic fragments. As a result, multiple marker ions with approximately the same RTs could correspond to a single PPD-quinone during the search stage. Molecules obtained by overlapping different screening approaches would give higher confidence levels for the identification of PPD-quinone compounds.

### Identification of Novel PPD-Quinones in an Environmental Sample

**Figure 4:**
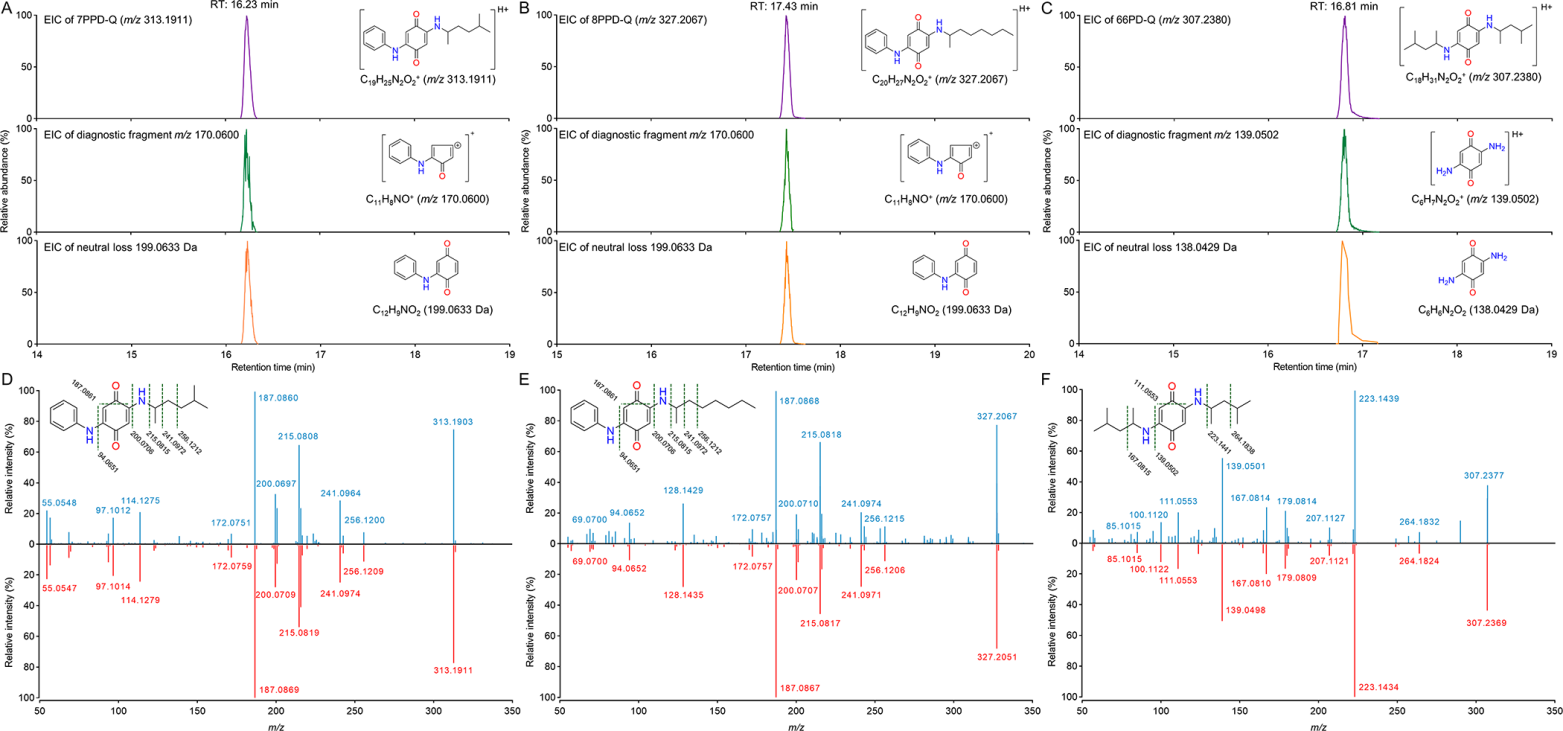
Identification of 7PPD-Q (A), 8PPD-Q (B), and 66PD-Q (C) with matched EICs of molecular ion, diagnostic fragment ions, and neutral losses (structures were attached with each MS transition). Each PPD-quinone was verified by comparing its MS^2^ spectra (upper) with the MS^2^ spectra of synthesized standards (lower) for 7PPD-Q (C), 8PPD-Q (D), and 66PD-Q (E). Structure elucidations were based on their respective MS^2^ spectra.

With the validated method, we conducted further analysis to screen potential PPD-quinone suspects in the environment. Multiple environmental samples, including tire tissue, air particulates, and surface soil, were analyzed. By conducting a crossover comparison of the NL ddMS^2^ transitions, DFF ions, and molecular screening with elemental composition restricted to the range C_1–40_H_0–100_N_2_O_2_ (general formula of PPD-quinone),^1,12−14^ a candidate list including nine ions was obtained (Table 1). Of those nine ions, six corresponded to previously reported PPD-quinones; these are ions with *m*/*z* 257.1285 at 12.75 min (IPPD-Q), *m*/*z* 291.1128 at 14.29 min (DPPD-Q), *m*/*z* 297.1598 at 15.14 min (CPPD-Q), *m*/*z* 319.1441 at 15.42 min (DTPD-Q), *m*/*z* 299.1754 at 15.57 min (6PPD-Q), and *m*/*z* 335.2693 at 18.38 min (77PD-Q), all of which were confirmed by RT matching to their standards. The remaining three ions were then checked for consistency in terms of the diagnostic fragment (i.e., *m*/*z* 170.0600, *m*/*z* 139.0502) and neutral loss (199.0633 and 138.0429 Da) transitions. Among them, *m*/*z* 313.1911 (Figure 4A) and *m*/*z* 327.2067 (Figure 4B) showed distinct high accordance with a DFF ion of *m*/*z* 170.0600 and NL of 199.0633 Da, while *m*/*z* 307.2380 (Figure 4C) exhibited a clear DFF ion of *m*/*z* 139.0502 and an NL of 138.0429. These results suggest that these candidates may be PPD-quinones, with *m*/*z* 313.1911 and *m*/*z* 327.2067 possibly belonging to Class I or II, and *m*/*z* 307.2380 being attributed to Class III. To further confirm this, these “suspect” ions were added to the PRM inclusion list to obtain their MS^2^ fingerprint spectra.

**Table 1:** Identified PPD-Quinone Candidates and Their Median Concentration among Different Environmental Samples (μg·g^–1^ for Tire Tissue, ng·g^–1^ in Surface Soil, pg·m^–3^ for Air Particulates)

compound	abbreviation	molecular formula	*m*/*z* [M + H]^+^	RT (min)	confidence level	tire tissue	air particles	surface soil
*N*-isopropyl-*N*′-phenyl-1,4-phenylenediamine quinone	IPPD-Q	C_15_H_16_N_2_O_2_	257.1285	12.75	Level 1	0.44	35.6	4.91
*N*,*N*′-diphenyl-*p*-phenylenediamine quinone	DPPD-Q	C_18_H_14_N_2_O_2_	291.1128	14.29	Level 1	1.59	22.6	9.75
*N*-phenyl-*N*′-cyclohexyl-*p*-phenylenediamine quinone	CPPD-Q	C_18_H_20_N_2_O_2_	297.1598	15.14	Level 1	1.66	12.2	2.85
*N*,*N*′-bis(methylphenyl)-1,4-benzenediamine quinone	DTPD-Q	C_20_H_18_N_2_O_2_	319.1441	15.42	Level 1	0.04	<LOQ	2.82
*N*-(1,3-dimethylbutyl)-*N*′-phenyl-*p*-phenylenediamine quinone	6PPD-Q	C_18_H_22_N_2_O_2_	299.1754	15.57	Level 1	13.7	34.9	141
*N*,*N*′-bis(1,4-dimethylpentyl)-*p*-phenylenediamine quinone	77PD-Q	C_20_H_34_N_2_O_2_	335.2693	18.38	Level 1	0.01	6.70	<LOQ
*N*-(1,4-dimethylpentyl)-*N*′-phenylbenzene-1,4-diamine quinone	7PPD-Q	C_19_H_24_N_2_O_2_	313.1911	16.23	Level 1	0.01	1.48	4.06
*N*-(1-methylheptyl)-*N*′-phenyl-1,4-benzenediamine quinone	8PPD-Q	C_20_H_26_N_2_O_2_	327.2067	17.43	Level 1	0.02	2.76	1.02
*N*,*N*′-di(1,3-dimethylbutyl)-*p*-phenylenediamine	66PD-Q	C_18_H_30_N_2_O_2_	307.2380	16.81	Level 1	0.21	0.40	0.23

**Figure 5:**
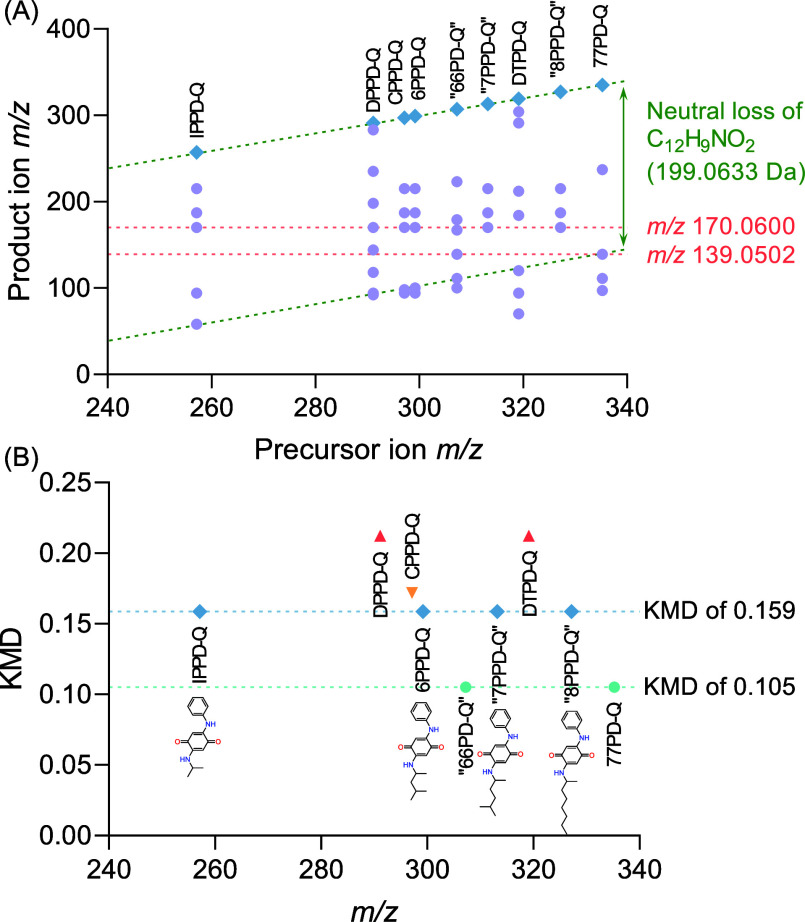
(A) Diagnostic fragmentation filtering plotting examples for PPD-quinones. Each point on a vertical line represents a PPD-quinone of the parent ion (blue diamond) and product ions (purple circle). (B) Kendrick mass defect diagram of PPD-quinones. Consistencies in the KMD of newly identified molecules are marked with lines. The structures of “6PPD-Q” class quinones (C_8_H_22_N_2_O_2_ ± (CH_2_)_*n*_, on blue dash line) are attached below. Newly identified PPD-quinones (i.e., 7PPD-Q, 8PPD-Q, 66PD-Q) are indicated in quotation marks.

MS^2^ spectra of *m*/*z* 313.1911 (Figure 4D) and *m*/*z* 327.2067 (Figure 4E) showed characteristic product ions of *m*/*z* 215.0815, *m*/*z* 187.0866, and *m*/*z* 170.0600, which are consistent with those of IPPD-Q, CPPD-Q, and 6PPD-Q. Additionally, the KMD diagram indicated that they fell into a horizontal line with IPPD-Q and 6PPD-Q (Figure 5). This combined evidence suggested that these compounds were a homologous series of PPD-quinones differing in a CH_2_ group. Moreover, this series of ions displayed increasing RTs with increasing *m*/*z* values. Considering that longer side alkyl chains may result in stronger hydrophobicity and, therefore, exhibit later RTs in a reversed-phase HPLC system, it is highly probable that these ions are congeners differing in the length of their side alkyl chains. This hypothesis was confirmed by their MS^2^ spectra, which showed clear neutral losses for the seven-membered (313.1900 → 215.0806, C_7_H_14_, 98.1096 Da) and eight-membered alkyl chains (327.2071 → 215.0806, C_8_H_16_, 112.1265 Da). Additionally, the corresponding aliphatic amine ions C_7_H_16_N^+^ (*m*/*z* 114.1277) and C_8_H_18_N^+^ (*m*/*z* 128.1434) were observed. This implies that *m*/*z* 313.1911 and *m*/*z* 327.2067 are PPD-quinones with alkyl side chains of seven and eight carbons, respectively. Given that PPD-quinones are the ozonation product of PPDs, we screened each parent compound for potential candidates by applying formulas that subtract two oxygen atoms and add two hydrogen atoms from these suspects’ formulas ([M] – O_2_ + H_2_). The results indicated that both PPDs (i.e., *N*-(1,4-dimethylpentyl)-*N*′-phenylbenzene-1,4-diamine, 7PPD and *N*-(1-methylheptyl)-*N*′-phenyl-1,4-benzenediamine, 8PPD) were detectable in the samples and well-matched with the standards. These comprehensive findings evidenced that *m*/*z* 313.1911 and *m*/*z* 327.2067 could be the quinone derivatives of PPDs, that is, 7PPD-Q and 8PPD-Q. To further validate this, we have synthesized the proposed structures of “7PPD-Q and 8PPD-Q” as described in the Supporting Information. It is worth noting that the synthesized standards exhibited the same RTs and MS^2^ spectra as those of 7PPD-Q and 8PPD-Q detected in environmental samples (Figure 4D,E). This finding marks the first documented occurrence of these contaminants.

Another suspect of *m*/*z* 307.2380 showed high consistency with the diagnostic fragment ion of *m*/*z* 139.0502 and the neutral loss of C_6_H_6_N_2_O_2_ (138.0429 Da, Figure 4C), implying it may possess symmetric alkyl substitution groups similar to those of 77PD-Q. The KMD diagram also confirmed this, with *m*/*z* 307.2380 sharing the same KMD of 0.105 with 77PD-Q. By further deducing its structure based on its MS^2^ fragments, we have observed the gradual losses of paired six-membered alkyl chain substitutions (307.2377 → 223.1441 → 139.0502, C_6_H_12_, 84.0938 Da) and corresponding fragment ion of aliphatic amine (C_6_H_14_N^+^, *m*/*z* 100.1120). By searching for the potential parent compound with the formula of C_18_H_32_N_2_ in *Scifinder*, we identified a *para*-phenylenediamine compound named *N*,*N*′-di(1,3-dimethylbutyl)-*p*-phenylenediamine (66PD), which has matching substitution groups (two 2-methylpentyl). Thus, it can be reasonably inferred that *m*/*z* 307.2380 was the quinone derivative of 66PD, i.e., 66PD-Q. It is reported that 66PD is applied as a tire rubber additive, making it reasonable for it to be transformed into 66PD-Q like other PPDs.^43^ To further explore this issue, we synthesized the standard chemical of 66PD-Q and parallelly compared their RTs and fragmentations. As depicted in Figure 4F, *m*/*z* 307.2380 matched perfectly with the synthesized standard, confirming that it is the novel PPD-quinone of 66PD-Q. Similar to 8PPD-Q, 66PD-Q was also identified, the structure elucidated, and finally confirmed for the first time in this study. As the identified novel PPD-quinones were all verified against synthesized standards through MS, MS^2^, and RTs, their identification can be classified as level 1 according to the Schymanski scale (Table 1).^44^

By summarizing the known and newly identified PPD-quinones with diagnostic fragmentation filtering plotting (Figure 5A), the feasibility of the developed approach based on featured fragments and neutral losses was proved, as all of the selected criteria are identifiable in their MS fragments plotting. Besides that, PPD-quinones, including IPPD-Q, 6PPD-Q, 7PPD-Q, and 8PPD-Q, are identified to be congeners that share the same KMD of 0.159, revealing their existence in series. These compounds show a remarkable regularity between their side alkyl chain length and their polarity; that is, a longer alkyl substitute leads to lower polarity, as indicated by their calculated Log *K*_ow_ values: 8PPD-Q (5.03) > 7PPD-Q (4.47) > 6PPD-Q (3.98) > IPPD-Q (2.58) (Table S3). The predicted values of Log *K*_ow_ are for reference only; estimations of their physicochemical properties are valuable as possible disagreement may occur between the measured and predicted values.^45^ In addition, featured fragmentations were observed for 6PPD-Q, 7PPD-Q, and 8PPD-Q through a comparison of their MS^2^ spectra (Figure S9). Identical fragments, including *m*/*z* 170.0600, 187.0866, 215.0815, indicate their shared *p*-phenylenediamine skeleton, while typical fragments such as *m*/*z* 100.1121 (C_6_H_14_N^+^) for 6PPD-Q, *m*/*z* 114.1277 (C_7_H_16_N^+^) for 7PPD-Q, and *m*/*z* 128.1434 (C_8_H_18_N^+^) for 8PPD-Q, reflect the unique structural of their alkyl chains of varying lengths. Also, the observation of such fragments confirmed the fragmentation pattern that we summarized (Figure 2). MS^2^ spectra of newly identified 7PPD-Q, 8PPD-Q, and 66PD-Q, obtained at different collision energies, are presented in Figures S10–S12 to facilitate future studies on the fragmentation patterns of PPD-quinones.

### Environmental Occurrences

Besides identification, we have also conducted further investigations to explore the occurrence of these PPD-quinones and their parent PPDs in multiple compartments, including tire tissue and ambient environmental matrices (i.e., air particulates and surface soil). Their median levels are summarized in Table 1, while their detailed detection frequencies and concentration ranges are given in Table S4. It is notable that the newly discovered 7PPD-Q, 8PPD-Q, and 66PD-Q are detected and quantified in air particulates and surface soil for the first time. Of the nine targeted PPD-quinones, 6PPD-Q demonstrated high detection frequency (98%) and environmental concentrations, with median levels of 13.7 μg·g^–1^ in tire tissue, 34.9 pg·m^–3^ in air particulates, and 141 ng·g^–1^ in surface soil. Comparatively, DPPD-Q was detected with a similar frequency (96%) but lower median levels (1.59 μg·g^–1^ in tire tissue, 22.6 pg·m^–3^ in air particulates, and 9.75 ng·g^–1^ in surface soil). 7PPD-Q and 8PPD-Q exhibited relatively lower levels in tire tissue (0.01, 0.02 μg·g^–1^ respectively) and air particulates (1.48, 2.76 pg·m^–3^ respectively). However, their levels in surface soil (4.06, 1.02 ng·g^–1^ respectively) were comparable to those of other PPD-quinones, including IPPD-Q (4.91 ng·g^–1^), DPPD-Q (9.75 ng·g^–1^), CPPD-Q (2.85 ng·g^–1^), and DTPD-Q (2.82 ng·g^–1^). 66PD-Q had higher concentrations in tire tissue (0.21 μg·g^–1^) but lower levels in surface soil (0.23 ng·g^–1^) and air particulates (0.40 pg·m^–3^) compared to those of 7PPD-Q and 8PPD-Q. Such findings indicate an assorted compositional profile of PPD-quinones among different environmental compartments. It is noteworthy that 7PPD-Q was previously detected in tire wear particles and recycled rubber products at a median level of 0.077 μg·g^–1^,^14^ which is comparable to our measurement in tire tissue (0.01 μg·g^–1^). These results collectively indicate the widespread occurrence of such contaminants in rubber-related products. The detection of these contaminants in the ambient environment compartments (e.g., air particulates and surface soil) may also imply their transportation from industrial products to the surrounding environments.

To further investigate the environmental characteristics of PPD-quinones, Spearman correlation analysis was performed between the newly identified and known PPD-quinones (Table S5). Significant correlations were found between 7PPD-Q, 8PPD-Q, and 66PD-Q, and these components also showed good correlations with 6PPD-Q (*R* = 0.56, 0.61, 0.72, respectively, *p* < 0.05), implying that these quinone contaminants share similar sources. Additionally, significant correlations were found between the PPD-quinones and their parent PPDs, with correlation coefficients *R*_7PPD/7PPD-Q_ = 0.37 (*p* < 0.05), *R*_8PPD/8PPD-Q_ = 0.59 (*p* < 0.05), *R*_66PD/66PD-Q_ = 0.47 (*p* < 0.05) being observed. It is reported that 7PPD is commonly used in combination with 6PPD, in a typical ratio of 2:1 or 1:1, as a rubber antioxidant to resist thermal and ozone aging.^46^ 8PPD can be dissolved in wax and added primarily to the tire sidewall at a rate of 3–7 per 100 parts by weight of a rubber component by some manufacturers.^47^ It is also applicable to rubber products bearing static and dynamic stress and subject to weather aging, such as tires, automotive door and window sealing strips, wires and cables, hoses, and tapes, due to its excellent protective effect against ozone aging cracks and flex cracks. The similarities regarding the production and utilization of PPDs may result in analogous environmental characteristics for their derived PPD-quinone contaminants.

### Environmental Implications

In nontargeted environmental sample analysis, identifying trace contaminants dispersed in complex matrices is a challenge akin to finding a needle in the proverbial haystack.^24,30^ Characteristic fragment filtering is a practical approach that uses marker ions to identify several “needles” (i.e., three novel PPD-quinones) in the “haystack.” In this study, we proposed a comprehensive strategy combining Full MS/AIF/NL ddMS^2^ scans to screen for both known and unknown PPD-quinone contaminants by integrating diagnostic fragmentation filtering and neutral loss scanning techniques. This strategy facilitated the detection of six known PPD-quinones and the identification of three novel PPD-quinones in multiple environmental matrices, including tire tissue, air particulates, and surface soil. Complete structural characterization and confirmation were achieved for the newly identified PPD-quinones with self-synthesized standards to match their RTs and MS/MS spectra. Meanwhile, using these standards, the composition and distribution profiles of nine PPD-quinones, as well as their parent PPDs, were further ascertained, in which the median levels of newly identified 7PPD-Q, 8PPD-Q, and 66PD-Q ranged from 0.01 to 0.21 μg·g^–1^ in tire tissue, 0.40–2.76 pg·m^–3^ in air particulates, and 0.23–4.06 ng·g^–1^ in surface soil. These findings broaden current knowledge about the presence of emerging PPD-quinone contaminants in the environment. Besides that, 8PPD-Q has branched alkyl side chains with eight carbon atoms and can be expected to be more lipophilic than 7PPD-Q and 6PPD-Q, like their parent PPDs.^48^ Assessment of its bioaccumulation and health risks would be valuable for further understanding its ecological impacts. Given the consistencies in the MS fragmentation behaviors and product ions of the TPs of PPD-quinones,^49,50^ potential applications using our proposed method to screen and identify these TPs are expected. This work exemplifies the screening of emerging environmental contaminants using a fragmentation pattern-based MS approach, which is anticipated to be expanded for the identification and quantitative analysis of various emerging contaminants, especially transformation chemicals, in the environment.

## Supporting Information Available

Synthesis and characterization of newly identified 7PPD-Q, 8PPD-Q, and 66PD-Q (SI 1); data calculation (SI 2); instrument parameters; additional QA/QC for quantifications; physicochemical properties of the PPD-quinones; measured concentration of PPDs and PPD-quinones in the environment and their correlations; MS^2^ spectra of known PPD-quinones with NCE of 10–60%; optimization of the all ion fragmentation collision energy; method validation with neutral loss fragmentation pattern; comparative MS^2^ spectra of 6PPD-Q with newly identified 7PPD-Q and 8PPD-Q; MS^2^ spectra of newly identified PPD-quinones with NCE of 10–60% (PDF)
