# Supplementary material for: Fragmentation Pattern-Based Screening Strategy Combining Diagnostic Ion and Neutral Loss Uncovered Novel para-Phenylenediamine Quinone Contaminants in the Environment
Source: Environ Sci Technol. 2024 Mar 21;58(13):5921–31. doi: 10.1021/acs.est.4c00027 (PMC10993393; doi:10.1021/acs.est.4c00027)
Supplement: Supplementary file 1 — es4c00027_si_001.pdf [file es4c00027_si_001.pdf]

Supporting Information

Fragmentation Pattern-Based Screening Strategy Combining Diagnostic Ion and Neutral Loss Uncovered Novel *para*-Phenylenediamine Quinone Contaminants in the Environment

Wei Wang[a], Guodong Cao[a], Jing Zhang[a], Weixia Chang[a], Yuecheng Sang[a] and Zongwei Cai[a, *]

[a] *State Key Laboratory of Environmental and Biological Analysis, Department of Chemistry, Hong Kong Baptist University, Hong Kong SAR 999077, China*

[*] Corresponding author: Zongwei Cai, email: zwcai@hkbu.edu.hk

Number of pages: 24

Number of figures: 12

Number of tables: 5

Contents

Text S1. Synthesis and characterization of 7PPD-Q, 8PPD-Q, and 66PD-Q.................................S5

Text S2. Additional information of data processing...S6

Table S1. Instrument parameters used in the identification and quantification of the analytes S7

Table S2. Optimized MRM parameters, recoveries, LODs and LOQs of the analytes...................S8

Table S3. Physicochemical properties of the PPD-quinones estimated by EPI Suite software S9

Table S4. Measured concentration of PPDs and PPD-quinones in the environment S10

Table S5. Spearman correlation of PPDs and PPD-quinones in the environment S11

Figure S1. MS^2 spectra of IPPD-Q with normalized HCD collision energy (NCE) of 10-60% ...S12

Figure S2. MS^2 spectra of DPPD-Q with normalized HCD collision energy (NCE) of 10-60%..S13

Figure S3. MS^2 spectra of CPPD-Q with normalized HCD collision energy (NCE) of 10-60%..S14

Figure S4. MS^2 spectra of 6PPD-Q with normalized HCD collision energy (NCE) of 10-60%...S15

Figure S5. MS^2 spectra of DTPD-Q with normalized HCD collision energy (NCE) of 10-60% .S16

Figure S6. MS^2 spectra of 77PD-Q with normalized HCD collision energy (NCE) of 10-60%...S17

Figure S7. Optimization of the all ion fragmentation collision energy....................................S18

Figure S8. Method validation with the recognition of each PPD-quinone.................................S19

Figure S9. Comparative MS^2 spectra of 6PPD-Q with 7PPD-Q and 8PPD-Q.........................S20

Figure S10. MS^2 spectra of 7PPD-Q with normalized HCD collision energy (NCE) of 10-60%.S21

Figure S11. MS^2 spectra of 8PPD-Q with normalized HCD collision energy (NCE) of 10-60%.S22

Figure S12. MS^2 spectra of 66PD-Q with normalized HCD collision energy (NCE) of 10-60%.S23

References...S24

Text S1. Synthesis and characterization of *N*-(1,4-dimethylpentyl)-*N′*-phenylbenzene-1,4-diamine quinone (7PPD-Q), *N*-(1-Methylheptyl)-*N′*-phenyl-1,4-benzenediamine quinone (8PPD-Q), and *N,N*'-di(1,3-dimethylbutyl)-*p*-phenylenediamine quinone (66PD-Q).

The synthesis reaction follows our early works,[1, 2] with its generally pathways are as followed:

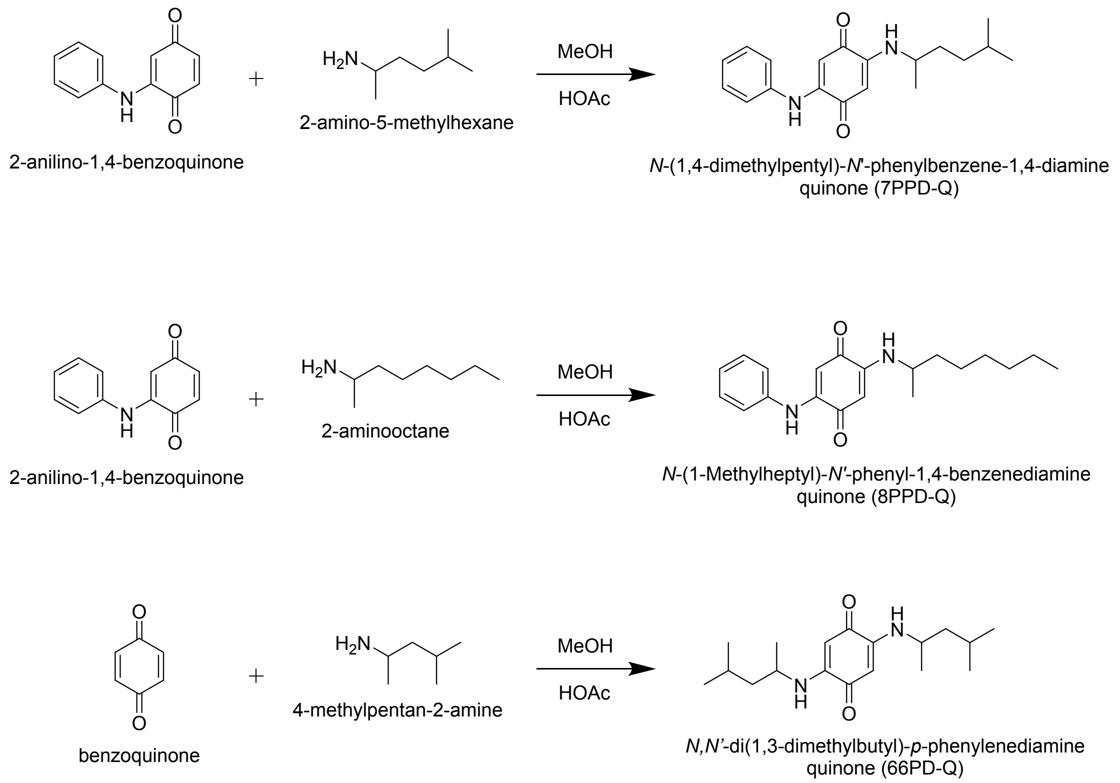

The synthesis of *N*-(1,4-dimethylpentyl)-*N′*-phenylbenzene-1,4-diamine quinone (7PPD-Q), *N*-(1-methylheptyl)-*N*'-phenyl-1,4-benzenediamine quinone (8PPD-Q), and *N,N*'-di(1,3-dimethylbutyl)-*p*-phenylenediamine quinone (66PD-Q) was conducted following the aforementioned scheme. For the synthesis of 7PPD-Q, we added 2-anilino-1,4-benzoquinone (500 mg, 2.5 mmol), 2-amino-5-methylhexane (324.4 mg, 2.5 mmol), methanol (10 mL) and ethanoic acid (216.3 μL) into a 25 mL round-bottomed flask. For 8PPD-Q, the materials were the same except the amine was replaced with 2-aminooctane (289.2 mg, 2.5 mmol) and the volume of ethanoic acid was adjusted to 253.7 μL. For 66PD-Q, the reaction substrate was benzoquinone (500 mg, 4.63 mmol) instead of 2-anilino-1,4-benzoquinone. We added 4-methylpentan-2-amine (314.8 mg, 3.08 mmol) and ethanoic acid (187.2 μL) to the round-bottomed flask, diluting with methanol (10 mL). The mixture was stirred at room temperature for 4 h. When the reaction was completed, 10 mL of water was added, and the precipitate was filtered and washed with water. The crude product was

purified by chromatography (*n*-hexane/dichloromethane = 1:1 to 2:3) to afford 7PPD-Q (64 mg, 0.20 mmol), 8PPD-Q (86 mg, 0.28 mmol), and 66PD-Q (32 mg, 0.10 mmol).

The characterizations of 7PPD-Q follow as: **^{1}H NMR** (400 MHz, Chloroform-*d*) δ 8.25 (s, 1H), 7.46-7.38 (m, 2H), 7.32-7.20 (m, 3H), 6.44 (d, *J* = 8.7 Hz, 1H), 5.99 (s, 1H), 5.43 (s, 1H), 3.46 (dh, *J* = 8.6, 6.5 Hz, 1H), 1.64-1.46 (m, 3H), 1.35-1.13 (m, 5H), 0.91 (dd, *J* = 6.7, 1.0 Hz, 6H). **^{13}C NMR** (101 MHz, Chloroform-*d*) δ 180.17, 178.49, 149.70, 147.70, 137.41, 129.73, 126.04, 122.84, 95.70, 93.00, 49.00, 35.17, 34.17, 28.13, 22.63 (d, *J* = 4.7 Hz), 19.92. **IR** (neat): 3265, 3233, 2954, 2868, 1639, 1557, 1487, 1440, 1354, 1288, 831, 769, 730, 692 cm^{-1}; **HR-ESI-MS**: *m/z* = 319.1900 $[C_{19}H_{25}N_2O_2]^+$ (calculated *m/z* = 313.1911). **HR-ESI-MS/MS** (*m/z*) 241.0962 $[M - C_5H_{12}]^+$ (calculated *m/z* = 241.0972), 215.0819 $[M - C_7H_{14}]^+$ (calculated *m/z* = 215.0815), 200.0709 $[M – C_7H_{15}N]^+$ (calculated *m/z* = 200.0706), 187.0869 $[M - C_8H_{14}O]^+$ (calculated *m/z* = 187.0866), 172.0757 $[M - C_8H_{15}NO]^+$ (calculated *m/z* = 172.0757), 114.1279 $[M - C_{12}H_9NO_2]^+$ (calculated *m/z* = 114.1277), 97.1014 $[M - C_{12}H_{12}N_2O_2]^+$ (calculated *m/z* = 97.1012),and 84.0446 $[M - C_{15}H_{19}NO]^+$ (calculated *m/z* = 84.0444).

The characterizations of 8PPD-Q follow as: **^{1}H NMR** (400 MHz, Chloroform-*d*): δ 8.24 (s, 1H), 7.46-7.35 (m, 2H), 7.30-7.18 (m, 3H), 6.42 (d, *J* = 8.6 Hz, 1H), 5.97 (s, 1H), 5.42 (s, 1H), 3.47 (dh, *J* = 8.5, 6.5 Hz, 1H), 1.57 (dddd, *J* = 20.0, 14.2, 7.2, 4.1 Hz, 2H), 1.38-1.21 (m, 11H), 0.92-0.85 (m, 3H). 1H NMR (400 MHz, Chloroform-d): **^{13}C NMR** (101 MHz, Chloroform-*d*): δ 180.13, 178.44, 149.68, 147.66, 137.38, 129.70, 126.01, 122.81, 95.67, 92.97, 48.71, 36.30, 31.82, 29.22, 26.10, 22.69, 19.88, 14.19. **IR** (neat) 3235, 2928, 2855, 1637, 1556, 1485, 1442, 1352, 1287, 1209, 829, 732, 691 cm^{-1}; **HR-ESI-MS**: *m/z* = 327.2051 $[C_{20}H_{27}N_2O_2]^+$ (calculated *m/z* = 327.2067). **HR-ESI-MS/MS** (*m/z*) 256.1206 $[M - C_5H_{11}]^+$ (calculated *m/z* = 256.1212), 241.0971 $[M - C_6H_{14}]^+$ (calculated *m/z* = 241.0972), 215.0817 $[M - C_8H_{16}]^+$ (calculated *m/z* = 215.0815), 200.0707 $[M - C_8H_{17}N]^+$ (calculated *m/z* = 200.0706), 187.0867 $[M - C_9H_{16}O]^+$ (calculated *m/z* = 187.0866), 172.0757 $[M - C_9H_{17}NO]^+$ (calculated *m/z* = 172.0757), 128.1435 $[M - C_{12}H_9NO_2]^+$ (calculated *m/z* = 128.1434) and 94.0652 $[M - C_{14}H_{19}NO_2]^+$ (calculated *m/z* = 94.0651).

The characterizations of 66PD-Q follow as: **^{1}H NMR** (400 MHz, Chloroform-*d*): δ 6.51 (d, *J* = 8.8 Hz, 2H), 5.32 (s, 2H), 3.51 (ddq, *J* = 14.6, 8.2, 6.4 Hz, 2H), 1.64 (ddq, *J* = 13.0, 7.6, 6.5 Hz, 2H), 1.51 (ddd, *J* = 14.3, 7.8, 6.6 Hz, 2H), 1.35 (ddd, *J* = 13.9, 7.8, 6.2 Hz, 2H), 1.19 (d, *J* = 6.4 Hz, 6H), 0.89 (dd, *J* = 11.7, 6.6 Hz, 12H). **^{13}C NMR** (101 MHz, Chloroform-*d*): δ 178.10, 150.62, 92.51,

46.69, 45.56, 25.11, 22.57, 20.17. **IR** (neat) 3245, 2951, 1643, 1556, 1485, 1351, 1301, 1251, 1210, 1160, 1125, 1056, 979, 937, 849, 810, 735 cm^{-1}; **HR-ESI-MS**: *m/z* = 307.2369 $[C_{18}H_{31}N_2O_2]^+$ (calculated *m/z* = 307.2380). **HR-ESI-MS/MS** (*m/z*) 264.1824 $[M - C_3H_7]^+$ (calculated *m/z* = 264.1832), 223.1434 $[M - C_6H_{12}]^+$ (calculated *m/z* = 223.1441), 207.1121 $[M - C_7H_{16}]^+$ (calculated *m/z* = 207.1128), 179.0809 $[M - C_9H_{20}]^+$ (calculated *m/z* = 179.0815), 167.0810 $[M - C_{10}H_{20}]^+$ (calculated *m/z* = 167.0815), 139.0498 $[M - C_{12}H_{24}]^+$ (calculated *m/z* = 139.0502), 111.0553 $[M - C_{13}H_{24}O]^+$ (calculated *m/z* = 111.0553), 100.1122 $[M - C_{12}H_{17}NO_2]^+$ (calculated *m/z* = 100.1121) and 85.1015 $[M - C_{12}H_{18}N_2O_2]^+$ (calculated *m/z* = 85.1012).

Text S2. Additional information of data processing.

Statistical analysis including the calculation of the geometric mean, concentration range, median level of the analytes, and Spearman correlation were conducted using SPSS 11.0 (IBM, SPSS Inc.). Two-tailed test was utilized for Spearman correlation analysis and a p-value < 0.05 was considered statistically significant. The physicochemical properties of the natural form of PPD-quinones were estimated with EPI Suite software (V.4.11, US EPA).[3] The obtained molecules were based on the general formula of PPD-quinones and filtered using compositional elemental constrained as $C_{1–40}H_{0–100}O_2N_2$ and *m/z* tolerance was 10 ppm.[1, 2, 4, 5]

The Kendrick mass defect (KMD) was adopted to identify molecules with related compositions, and molecular formulas were sorted according to their differences in the homologous series of CH_2. Its calculation was based on the following equations:[6, 7]

$$KM = IUPAC\ mass \times (\frac{14.00000}{14.01565})$$

$$KMD = Nominal\ mass - KM$$

where *IUPAC mass* is the exact mass of a compound calculated from the *m/z* value measured by the mass spectrometer, while *Nominal mass* is the rounded integer mass of a compound.

Table S1. Instrument parameters used in the identification and quantification of the analytes.

Instrument	Parameter	Value
UPLC	Flow rate	300 μL·min^{-1}
	Injection volume	2 μL
	Column	Waters Acquity HSS T3 column (1.8 μm, 2.1×100 mm)
	Column temperature	35 ℃
	Mobile phase	(A) deionized water with 0.1% formic acid in (B) acetonitrile with 0.1% formic acid
	Solvent gradient	2% B for 1 min, increased linearly to 100% B in 19 min and hold for 3 min, decreased to 2% B in 0.1 min and hold for 4.9 min.
ESI-Q Orbitrap MS	Ionization mode	Positive ESI
	Vaporizer temperature	300 ℃
	Spray voltage	3500 V
	Scan range	80-500
	Sheath gas flow	45 arb
	Auxiliary gas flow	10 arb
	Cone gas	Nitrogen
	Collision gas	Argon
	Full MS resolution	35,000 FWHM
	ddMS^2 resolution	17,500 FWHM
	S-Lens RF level	55%
	AIF NCE	50%
	NL ddMS^2 NCE	10 20 40%
	PRM NCE	10 20 40%
ESI-TQ MS	Ionization mode	Positive ESI
	Vaporizer temperature	350 ℃
	Cone gas	Nitrogen
	Collision gas	Argon
	Collision energy	Specified for each analyte (Table S3)

Table S2. Optimized MRM parameters, recoveries (mean ± S.D., %), limits of detection (LODs) and limits of quantification (LOQs) of the analytes.[a]

Compound	Precursor ion	Quantifier product ion	Collision energy (eV)	Qualifier product ion	Tire tissue			Air particulate			Surface soil		
					Recovery (%)	LODs ($\mu g \cdot g^{-1}$)	LOQs ($\mu g \cdot g^{-1}$)	Recovery (%)	LODs ($pg \cdot m^{-3}$)	LOQs ($pg \cdot m^{-3}$)	Recovery (%)	LODs ($ng \cdot g^{-1}$)	LOQs ($ng \cdot g^{-1}$)
IPPD	227.2	184.1	26	212.1/168.1	85±12	0.002	0.004	88±8	0.078	0.141	105±18	0.10	0.18
CPPD	267.2	185.1	22	223.1/130.1	81±9	0.007	0.008	73±12	0.242	0.258	75±8	0.31	0.33
6PPD	269.2	93.1	32	184.1/212.1	106±17	0.004	0.008	93±9	0.141	0.273	90±12	0.18	0.35
DPPD	261.1	184.1	26	169.1/107.1	101±22	0.007	0.008	87±16	0.234	0.273	92±6	0.30	0.35
DTPD	289.2	198.1	23	183.1/106.1	98±12	0.012	0.014	94±14	0.398	0.469	77±14	0.51	0.60
66PD	277.3	100.1	32	193.2/176.1	-	-	-	-	-	-	-	-	-
77PD	305.3	206.2	16	233.2/135.1	73±11	0.018	0.023	67±3	0.602	0.781	83±11	0.77	1.00
7PPD	283.2	184.1	20	93.1/212.1	86±9	0.009	0.011	72±6	0.305	0.375	70±6	0.39	0.48
8PPD	297.2	185.1	20	211.1/281.2	82±8	0.009	0.017	70±12	0.289	0.555	64±14	0.37	0.71
IPPD-Q	257.1	187.1	24	215.1/172.1	117±20	0.014	0.018	92±5	0.469	0.617	97±13	0.60	0.79
CPPD-Q	297.2	187.1	28	215.1/98.1	87±13	0.004	0.011	88±9	0.148	0.367	90±8	0.19	0.47
6PPD-Q	299.2	241.1	26	215.1/187.1	105±11	0.002	0.007	83±7	0.078	0.250	96±11	0.10	0.32
DPPD-Q	291.1	263.1	20	235.1/144.1	72±12	0.004	0.009	91±9	0.148	0.305	88±6	0.19	0.39
DTPD-Q	319.1	184.1	27	212.1/301.1	77±13	0.007	0.011	81±11	0.234	0.359	70±4	0.30	0.46
66PD-Q	307.2	223.1	18	100.1/139.0	92±6	0.002	0.009	91±13	0.078	0.313	81±10	0.10	0.40
77PD-Q	335.2	237.1	18	139.0/97.1	96±10	0.009	0.011	90±8	0.289	0.359	85±8	0.37	0.46

7PPD-Q	313.2	187.1	28	215.1/114.1	103±9	0.008	0.009	87±6	0.273	0.305	96±14	0.35	0.39
8PPD-Q	327.2	187.1	30	215.1/128.1	98±12	0.007	0.011	83±11	0.234	0.367	76±18	0.30	0.47

[a] Limits of detection (LODs) and quantification (LOQs) were defined as the average plus 3- and 10-fold standard deviation (S.D.) of method blank levels, or analyte concentrations with signal-to-noise ratios of 3 and 10, whichever was higher.

Table S3. Physicochemical properties[a] of the PPD-quinones estimated by EPI Suite software.[3]

Compound	Water solubility[b] (mg/L, 25 ℃)	Log K_{ow}[c]	Log K_{oa}[d]	Log K_{oc}[e]	Log BCF[f]	Log BAF[g]
IPPD-Q	1400	2.58	14.288	3.165	1.367	1.359
DPPD-Q	15.38	3.46	16.014	3.652	1.953	1.258
CPPD-Q	56.93	3.94	15.635	3.581	2.265	2.206
DTPD-Q	1.225	4.56	17.028	4.260	2.675	2.363
6PPD-Q	51.34	3.98	15.319	3.939	2.291	2.120
7PPD-Q	16.18	4.47	15.685	4.210	2.615	2.291
8PPD-Q	4.402	5.03	16.122	4.520	2.987	2.490
66PD-Q	16.81	4.49	14.614	4.221	2.629	2.945
77PD-Q	1.662	5.47	15.348	4.763	3.277	3.432

[a] The predicted level were only for reference as there may be discrepancies between estimated and measured values.[8]

[b] Water solubility was estimate from Log K_{ow} (WSKOW v1.42).

[c] Log K_{ow}: octanol-water partition coefficient, estimated from KOWWIN v1.69.

[d] Log K_{oa}: octanol-air partition coefficient, estimate from KOAWIN v1.10.

[e] Log K_{oc}: organic carbon partition coefficient, estimate using K_{ow} method.

[f] Log BCF: bioconcentration factor estimated from regression-based method.

[g] Log BAF: bioaccumulation factor estimated from Arnot-Gobas method.

Table S4. Measured concentration of PPDs and PPD-quinones in tire tissue (μg·g^{-1}), air particulate (pg·m^{-3}), and surface soil (ng·g^{-1}).

Compound	Tire tissue (*N*=8)				Air particulate (*N*=20)				Surface soil (*N*=24)			
	DF[a]	Mean	Median	Range	DF	Mean	Median	Range	DF	Mean	Median	Range
PPDs												
IPPD	88	0.34	0.29	<LOQ[b]-0.44	85	1.85	1.62	<LOQ-5.53	50	1.29	0.09	<LOQ-9.88
DPPD	100	5.50	4.57	1.92-14.2	60	17.0	4.77	<LOQ-63.8	96	62.9	14.7	<LOQ-309
CPPD	100	4.07	3.39	0.98-7.30	30	1.78	<LOQ	<LOQ-12.2	46	1.16	<LOQ	<LOQ-5.73
DTPD	100	0.46	0.40	0.05-1.34	25	0.52	<LOQ	<LOQ-3.41	75	27.8	4.49	<LOQ-155
6PPD	100	344	272	239-502	95	1360	267	2.18-7150	100	195	41.1	3.03-1290
7PPD	88	0.08	0.08	<LOQ-0.14	35	1.48	<LOQ	<LOQ-15.9	33	0.53	<LOQ	<LOQ-3.00
8PPD	75	3.72	3.86	<LOQ-8.37	65	11.9	6.62	<LOQ-54.5	71	2.73	1.36	<LOQ-10.0
66PD[c]	75	0.53	0.24	<LOQ-2.24	50	0.70	<LOQ	<LOQ-4.37	54	0.73	<LOQ	<LOQ-8.37
77PD	25	0.09	<LOQ	<LOQ-0.45	35	1.93	<LOQ	<LOQ-7.20	21	40.9	<LOQ	<LOQ-1.84
PPD-Quinones												
IPPD-Q	75	0.43	0.44	<LOQ-0.93	90	70.3	35.6	<LOQ-515	71	67.7	4.91	<LOQ-922
DPPD-Q	100	1.82	1.59	0.97-2.68	95	193	22.6	2.27-2960	92	81.0	9.75	<LOQ-932
CPPD-Q	100	3.48	1.66	1.29-8.94	70	16.8	12.2	<LOQ-71.0	75	46.6	2.85	<LOQ-606
DTPD-Q	63	0.05	0.04	<LOQ-0.13	30	1.38	<LOQ	<LOQ-9.33	75	13.5	2.82	<LOQ-67.4
6PPD-Q	100	14.3	13.7	7.98-26.3	95	80.3	34.9	2.70-387	100	242	141	0.86-939
7PPD-Q	50	0.01	0.01	<LOQ-0.05	25	7.90	1.48	<LOQ-65.1	92	20.2	4.06	<LOQ-248
8PPD-Q	63	0.02	0.02	<LOQ-0.02	85	4.79	2.76	<LOQ-14.5	58	5.34	1.02	<LOQ-52.7
66PD-Q	100	0.53	0.21	0.15-1.56	60	3.28	0.40	<LOQ-16.3	92	1.97	0.23	<LOQ-11.9
77PD-Q	88	0.05	0.01	<LOQ-0.22	70	42.8	6.70	<LOQ-194	38	1.50	<LOQ	<LOQ-8.09

[a] DF = Detection frequency.

[b] LOQ = Limit of quantification.

[c] Concentration of 66PD is semi-quantified according to the standard calibration curve of 77PD.

Table S5. Spearman correlation coefficients (*R*) between concentrations of PPDs and PPD-quinones in tire tissue, air particulate, and surface soil.[a]

Compound	IPPD	DPPD	CPPD	DTPD	6PPD	7PPD	8PPD	66PD	77PD	IPPD-Q	DPPD-Q	CPPD-Q	DTPD-Q	6PPD-Q	7PPD-Q	8PPD-Q	66PD-Q	77PD-Q
IPPD	1.00																	
DPPD	0.54*	1.00																
CPPD	0.47*	0.43*	1.00															
DTPD	0.22	0.59*	0.53*	1.00														
6PPD	0.68*	0.76*	0.59*	0.34*	1.00													
7PPD	0.64*	0.55	0.45*	0.30*	0.64*	1.00												
8PPD	0.52*	0.18	0.38*	0.17	0.25	0.26*	1.00											
66PD	0.28*	0.24	0.45*	0.23	0.38*	0.21	0.19	1.00										
77PD	0.03	0.00	-0.13	-0.18	0.02	0.03	0.27	0.33*	1.00									
IPPD-Q	0.69*	0.53*	0.22	0.24	0.50*	0.38*	0.38*	0.09	0.14	1.00								
DPPD-Q	0.66*	0.49*	0.13	0.31*	0.38*	0.45*	0.38*	0.06	0.05	0.85*	1.00							
CPPD-Q	0.61*	0.69*	0.55*	0.52*	0.70*	0.47*	0.25	0.32*	-0.03	0.80*	0.68*	1.00						
DTPD-Q	0.28*	0.53*	0.47*	0.77*	0.29*	0.30*	0.21	0.21	-0.25	0.42*	0.49*	0.66*	1.00					
6PPD-Q	0.58*	0.76*	0.36*	0.69*	0.62*	0.54*	0.26	0.18	-0.02	0.68*	0.70*	0.79*	0.72*	1.00				
7PPD-Q	0.29*	0.46*	-0.01	0.34*	0.33*	0.37*	0.08	0.13	0.12	0.31*	0.45*	0.32*	0.39*	0.56*	1.00			
8PPD-Q	0.71*	0.51*	0.35*	0.26	0.50*	0.37*	0.59*	0.19	0.20	0.80*	0.75*	0.72*	0.43*	0.61*	0.30*	1.00		
66PD-Q	0.66*	0.68*	0.52*	0.43*	0.74*	0.61*	0.23	0.47*	0.02	0.62*	0.60*	0.77*	0.45*	0.72*	0.52*	0.57*	1.00	
77PD-Q	0.78*	0.46*	0.34*	0.04	0.67*	0.57*	0.29*	0.19	0.05	0.72*	0.67*	0.68*	0.14	0.52*	0.31*	0.66*	0.69*	1.00

[a] Two-tailed test was utilized.

* Significant at $p < 0.05$.

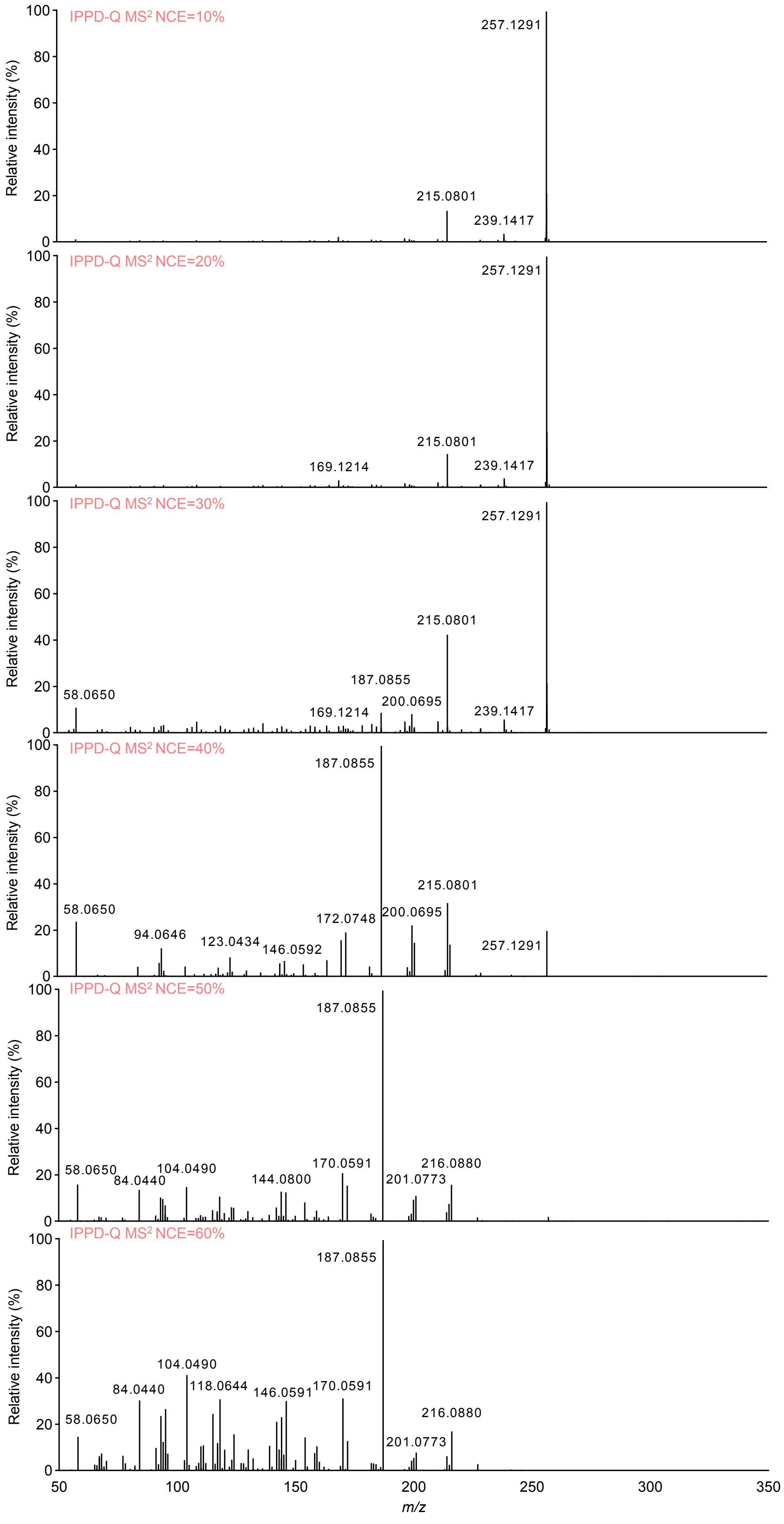

Figure S1. MS² spectra of IPPD-Q with normalized HCD collision energy (NCE) of 10-60%.

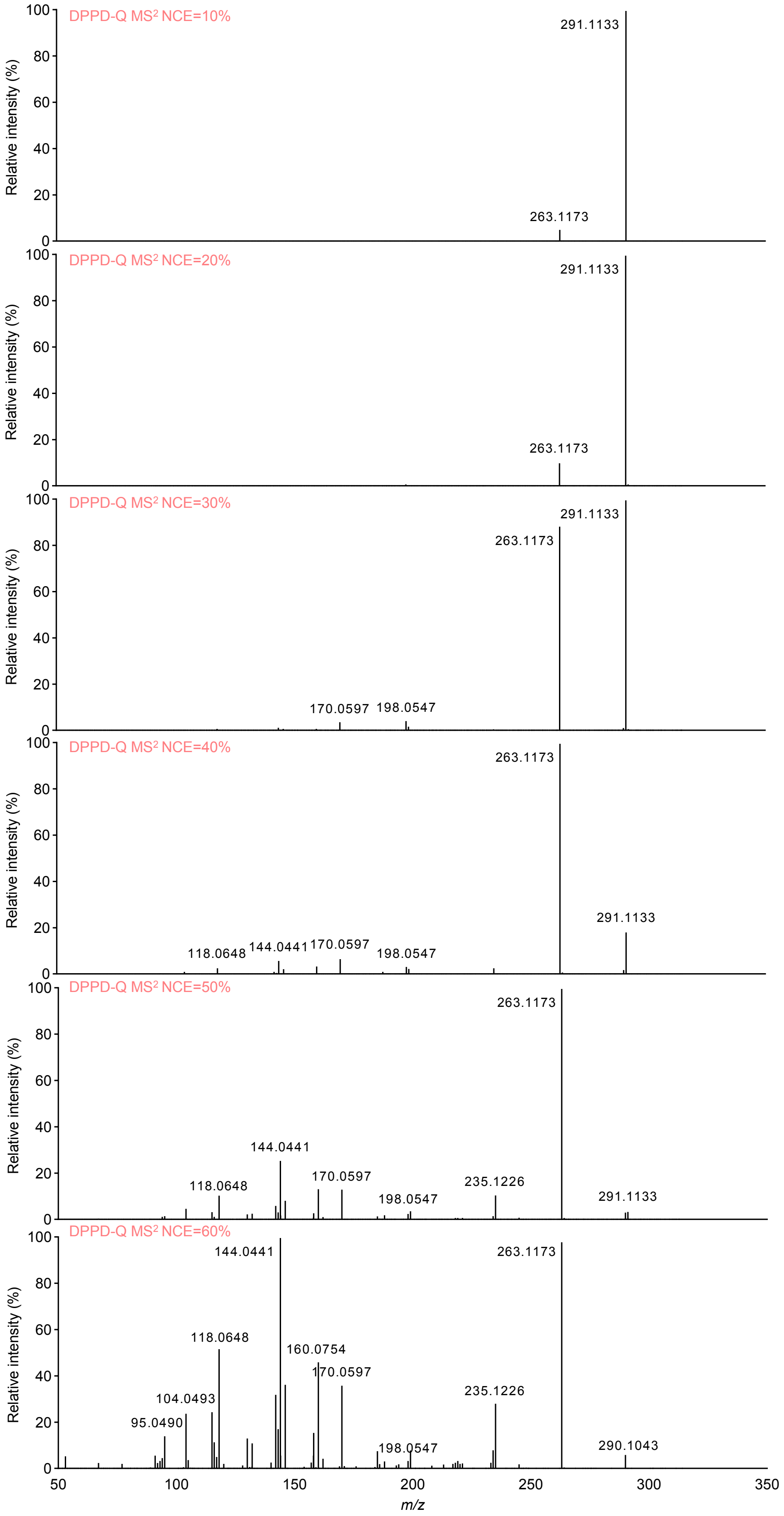

Figure S2. MS^2 spectra of DPPD-Q with normalized HCD collision energy (NCE) of 10-60%.

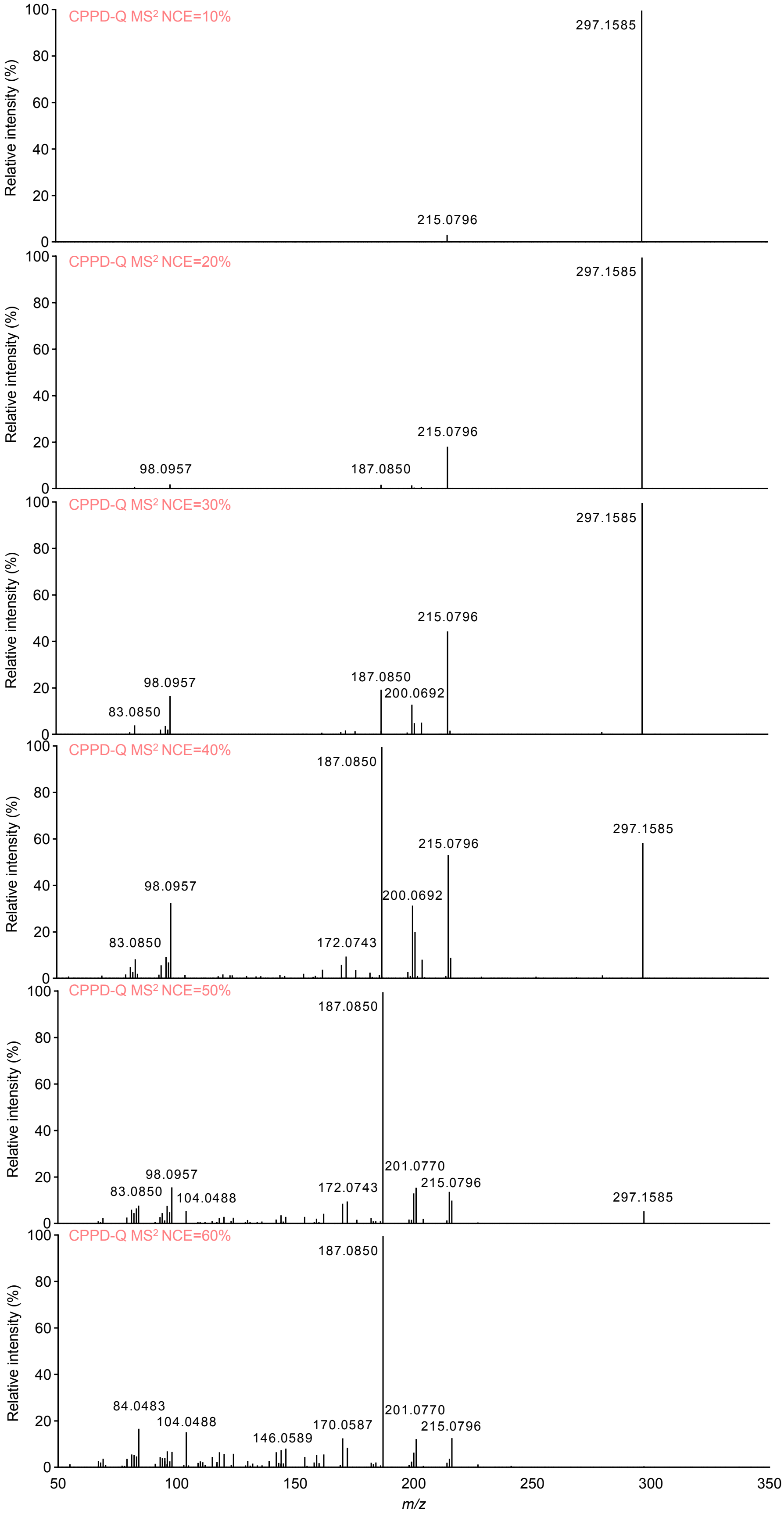

Figure S3. MS² spectra of CPPD-Q with normalized HCD collision energy (NCE) of 10-60%.

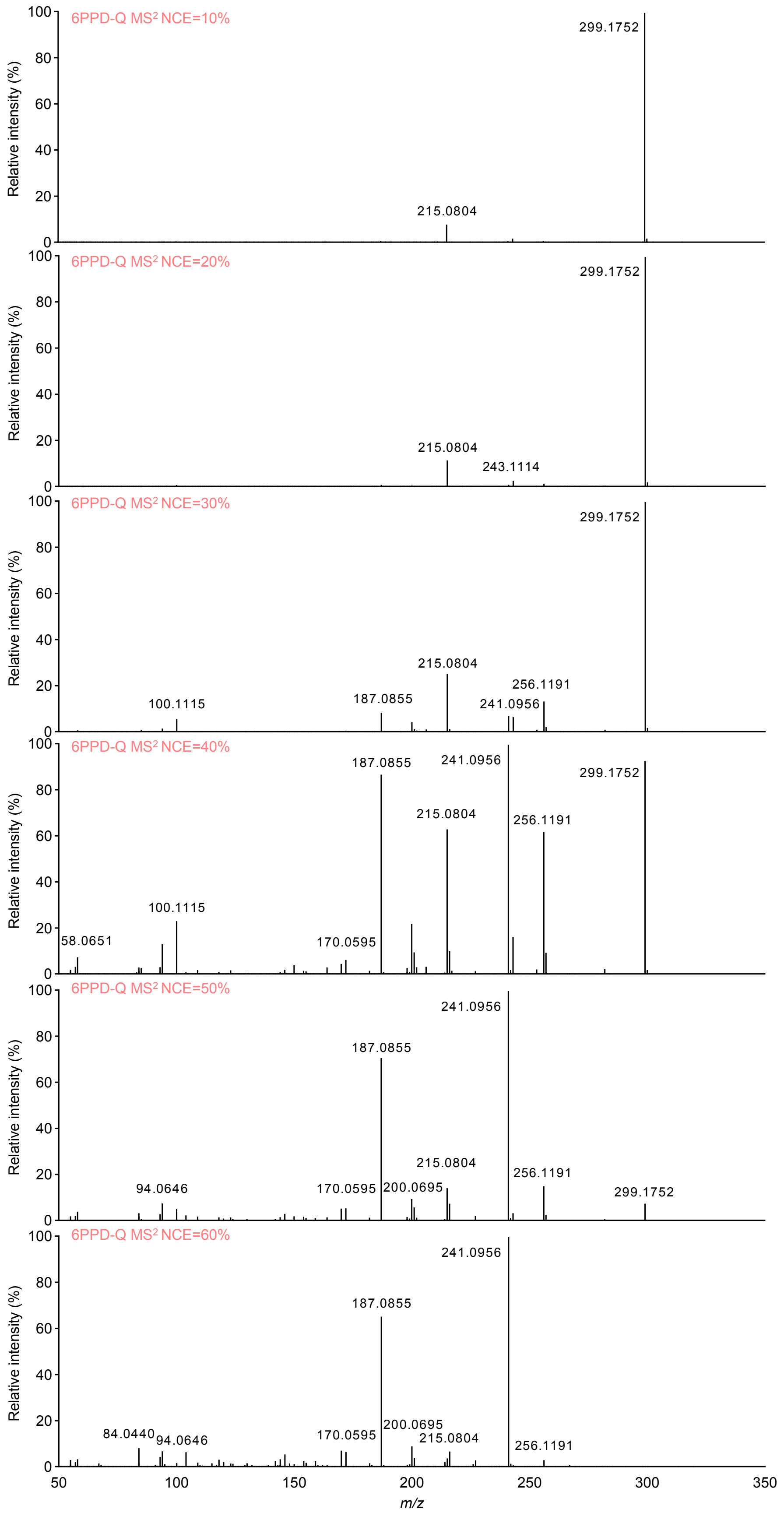

Figure S4. MS^2 spectra of 6PPD-Q with normalized HCD collision energy (NCE) of 10-60%.

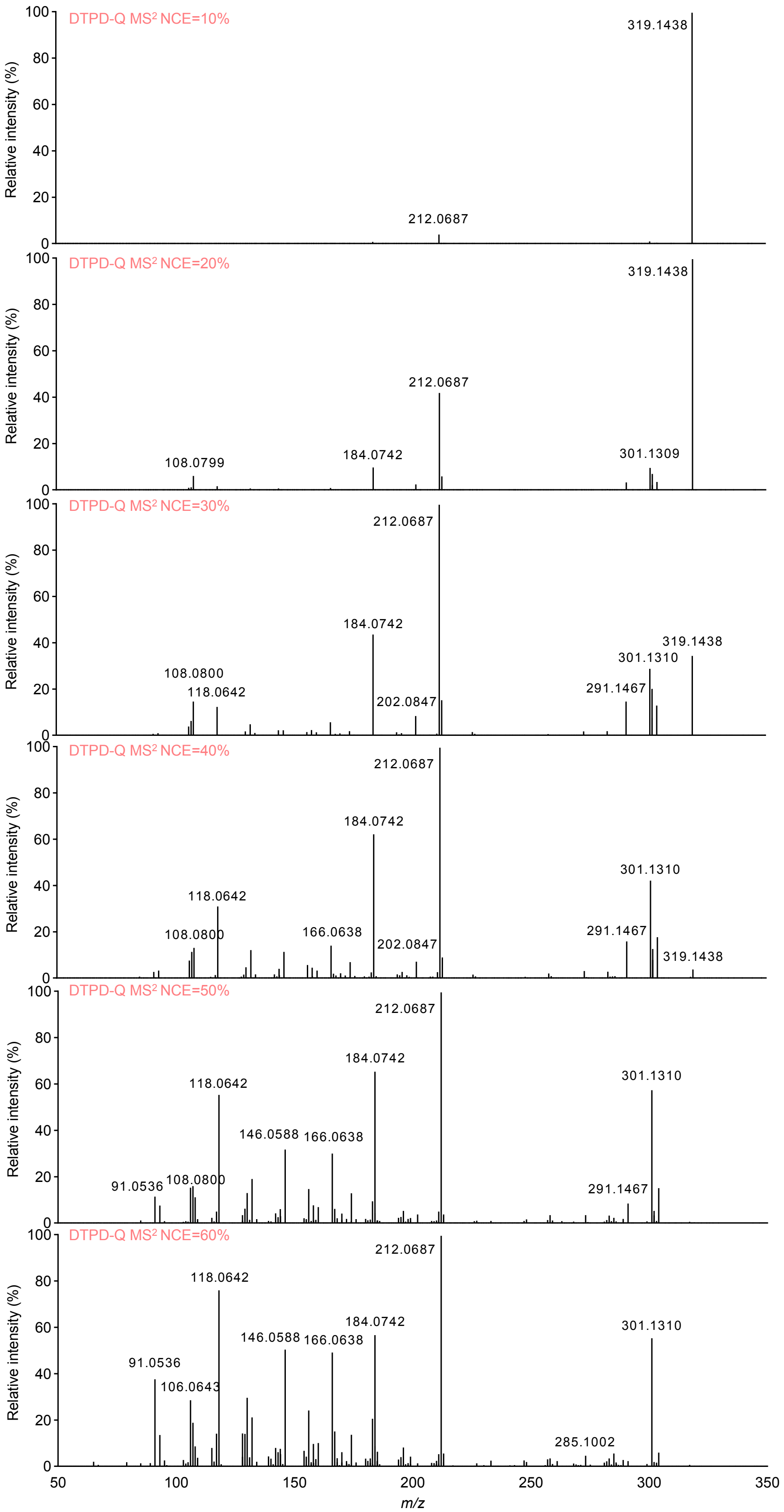

Figure S5. MS² spectra of DTPD-Q with normalized HCD collision energy (NCE) of 10-60%.

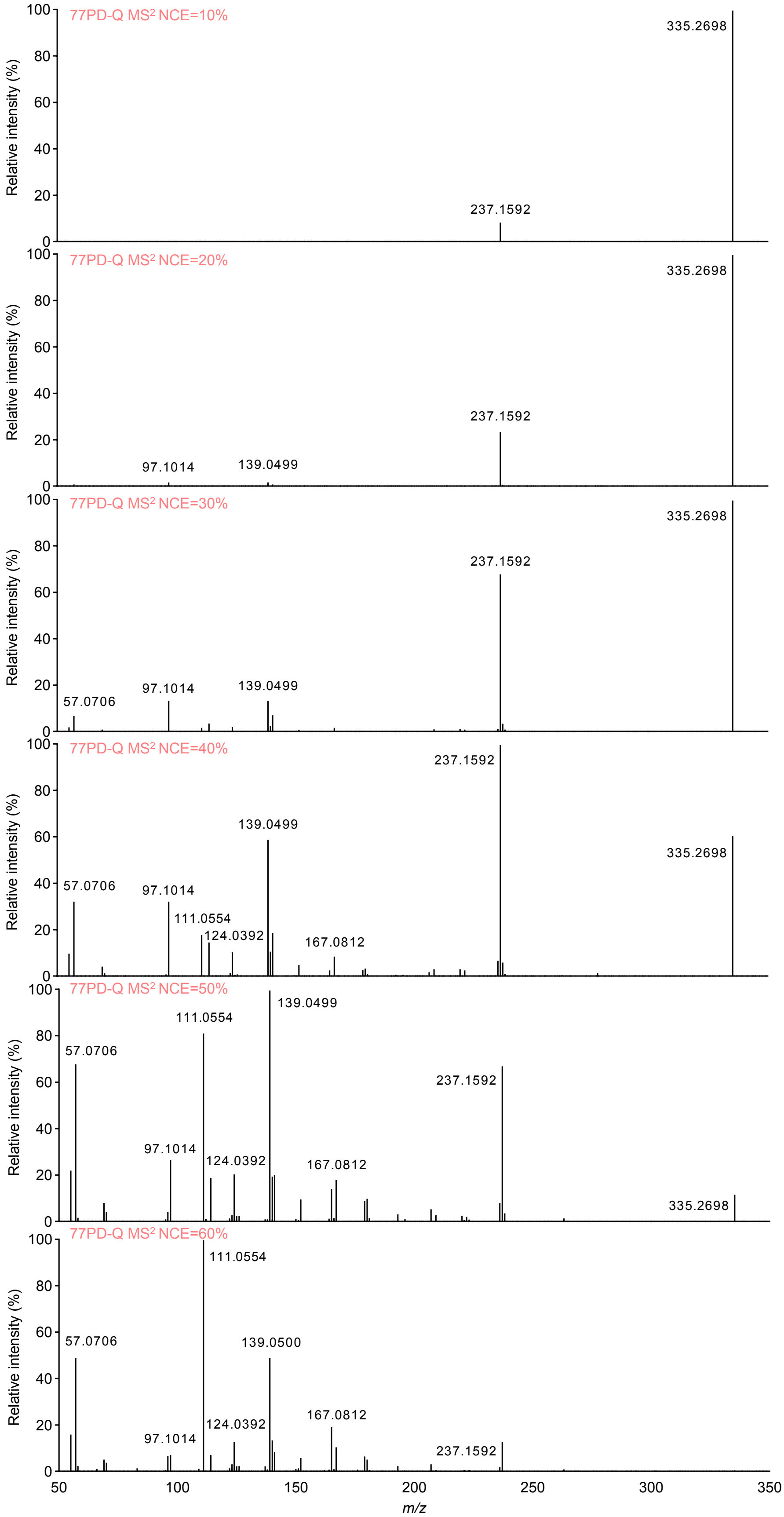

Figure S6. MS² spectra of 77PD-Q with normalized HCD collision energy (NCE) of 10-60%

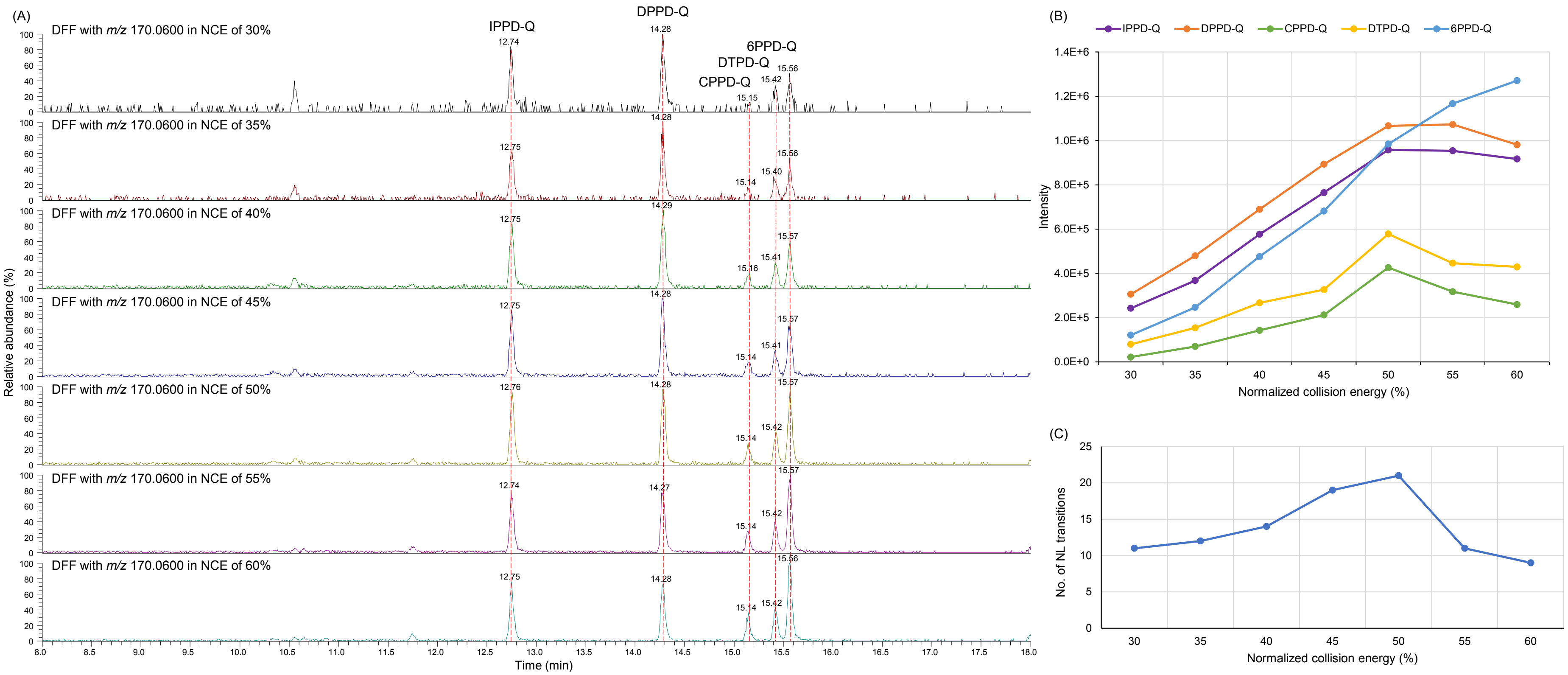

Figure S7. Optimization of the all ion fragmentation collision energy. (A) Chromatography of diagnostic fragment filtering with product ion of *m/z* 170.0600 in varying normalized collision energies. (B) Intensity of each PPD-quinone in different normalized collision energies. (C) Number of acquired neutral loss transitions in different normalized collision energies.

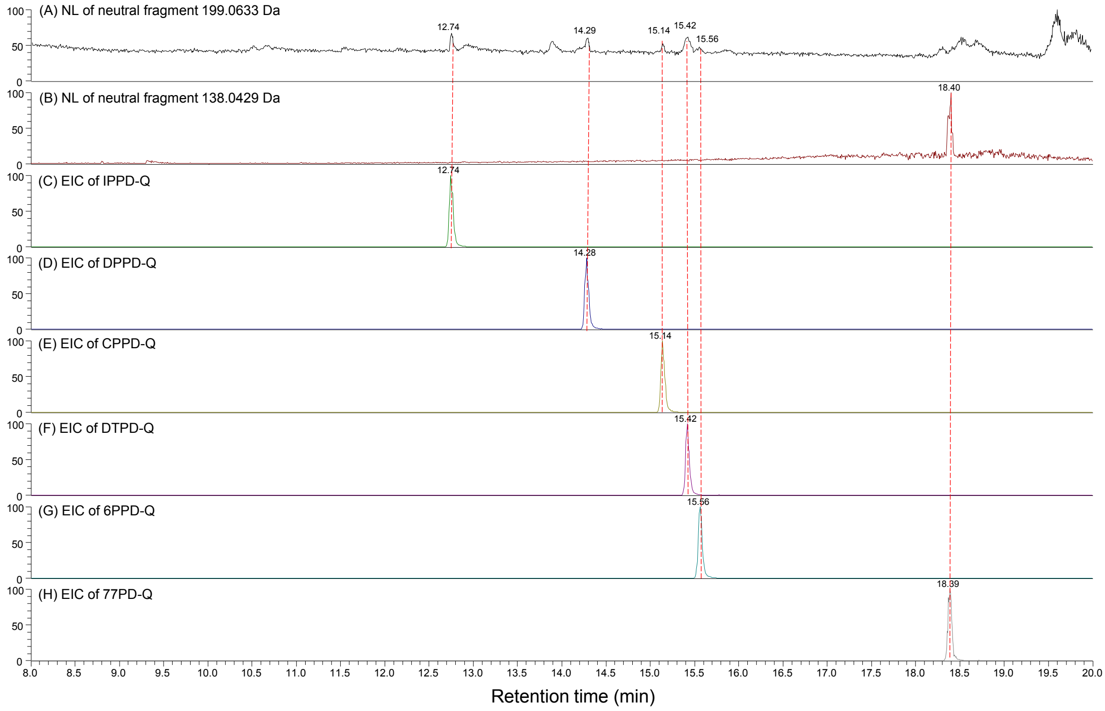

Figure S8. Method validation with the recognition of each PPD-quinone. Neutral loss scan with neutral fragment of $C_{12}H_9NO_2$ (199.0633 Da, A) was utilized to identify IPPD-Q (C), DPPD-Q (D), CPPD-Q (E), DTPD-Q (F), and 6PPD-Q (G), while neutral fragment of $C_6H_6N_2O_2$ (138.0429 Da, B) was adopted to identify 77PD-Q (H).

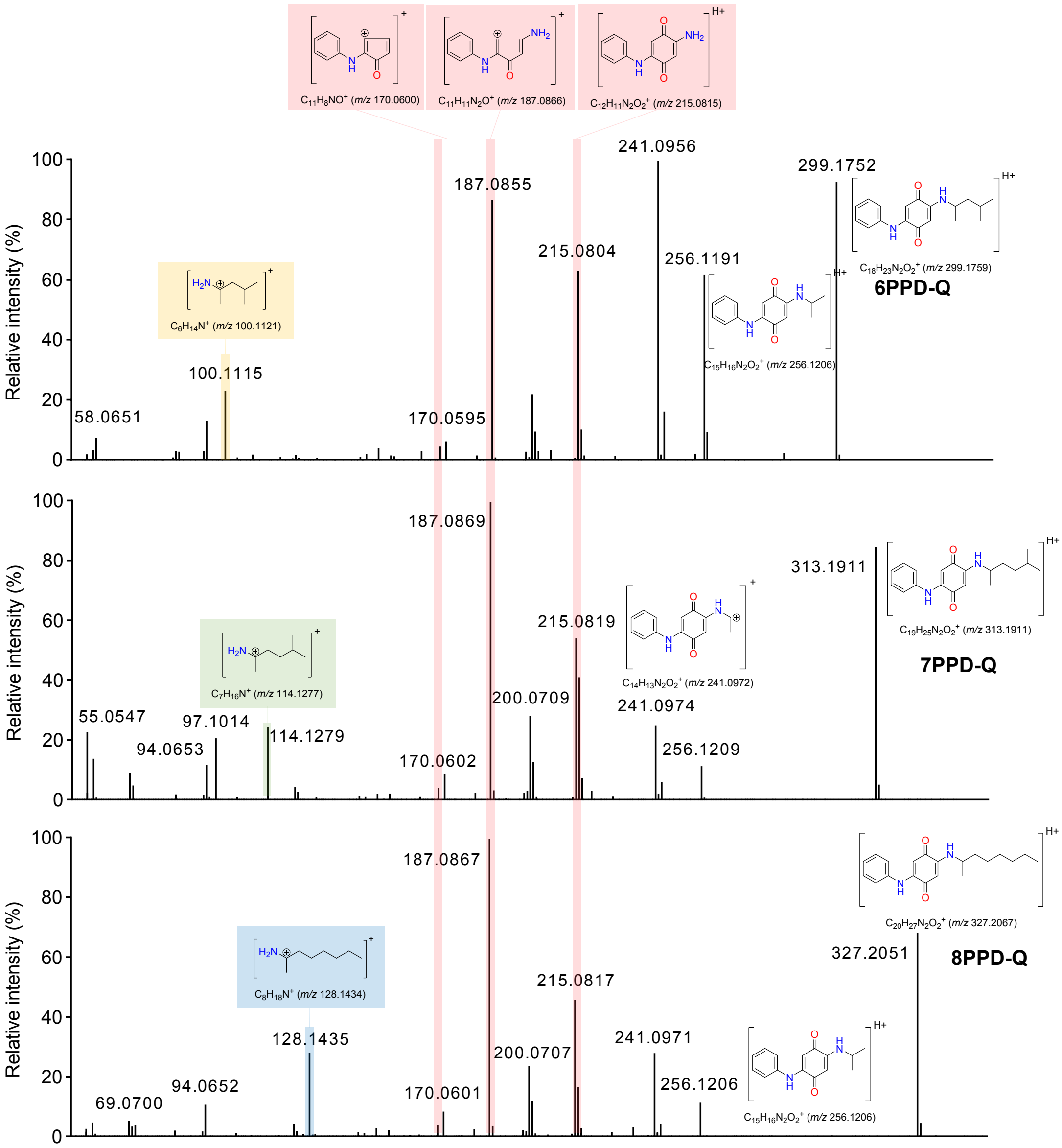

Figure S9. Comparative MS^2 spectra of 6PPD-Q with newly identified 7PPD-Q and 8PPD-Q. Identical fragments (*m/z* 170.0600, 187.0866, 215.0815) are marked in red, while typical fragments (*m/z* 100.1121 for 6PPD-Q, *m/z* 114.1277 for 7PPD-Q, and 128.1434 for 8PPD-Q) indicating their unique structural information are marked in different colors.

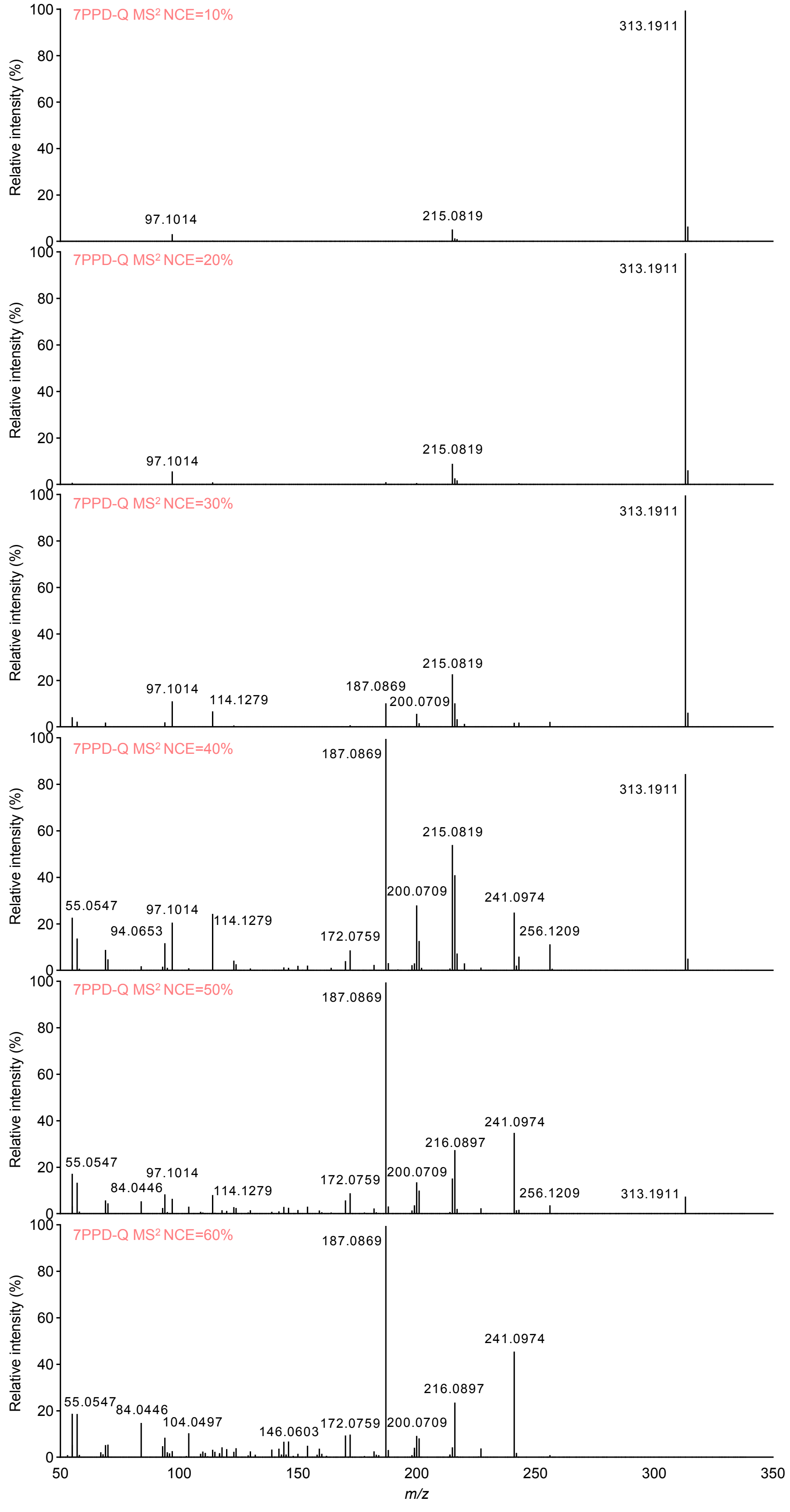

Figure S10. MS^2 spectra of 7PPD-Q with normalized HCD collision energy (NCE) of 10-60%

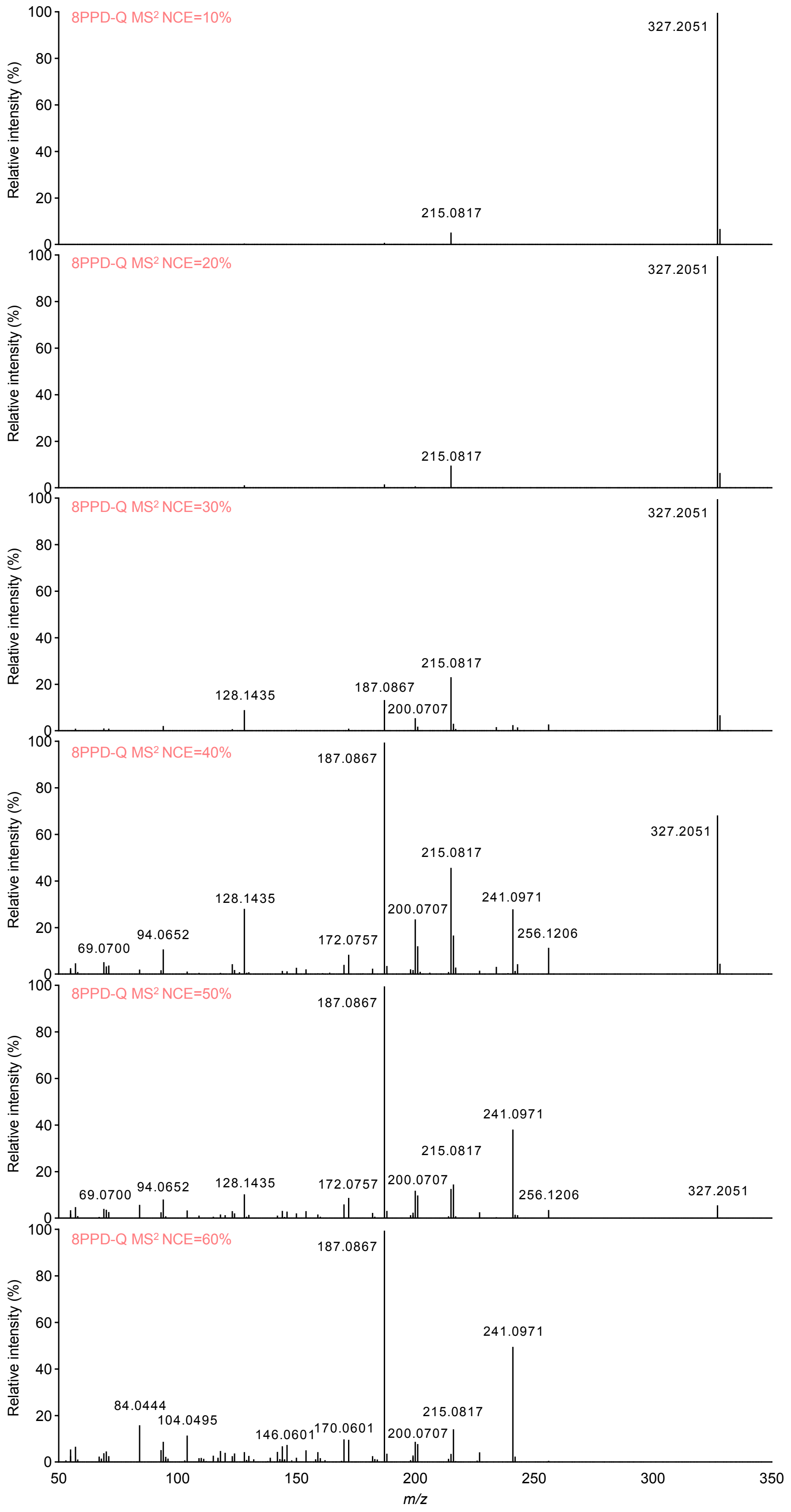

Figure S11. MS^2 spectra of 8PPD-Q with normalized HCD collision energy (NCE) of 10-60%

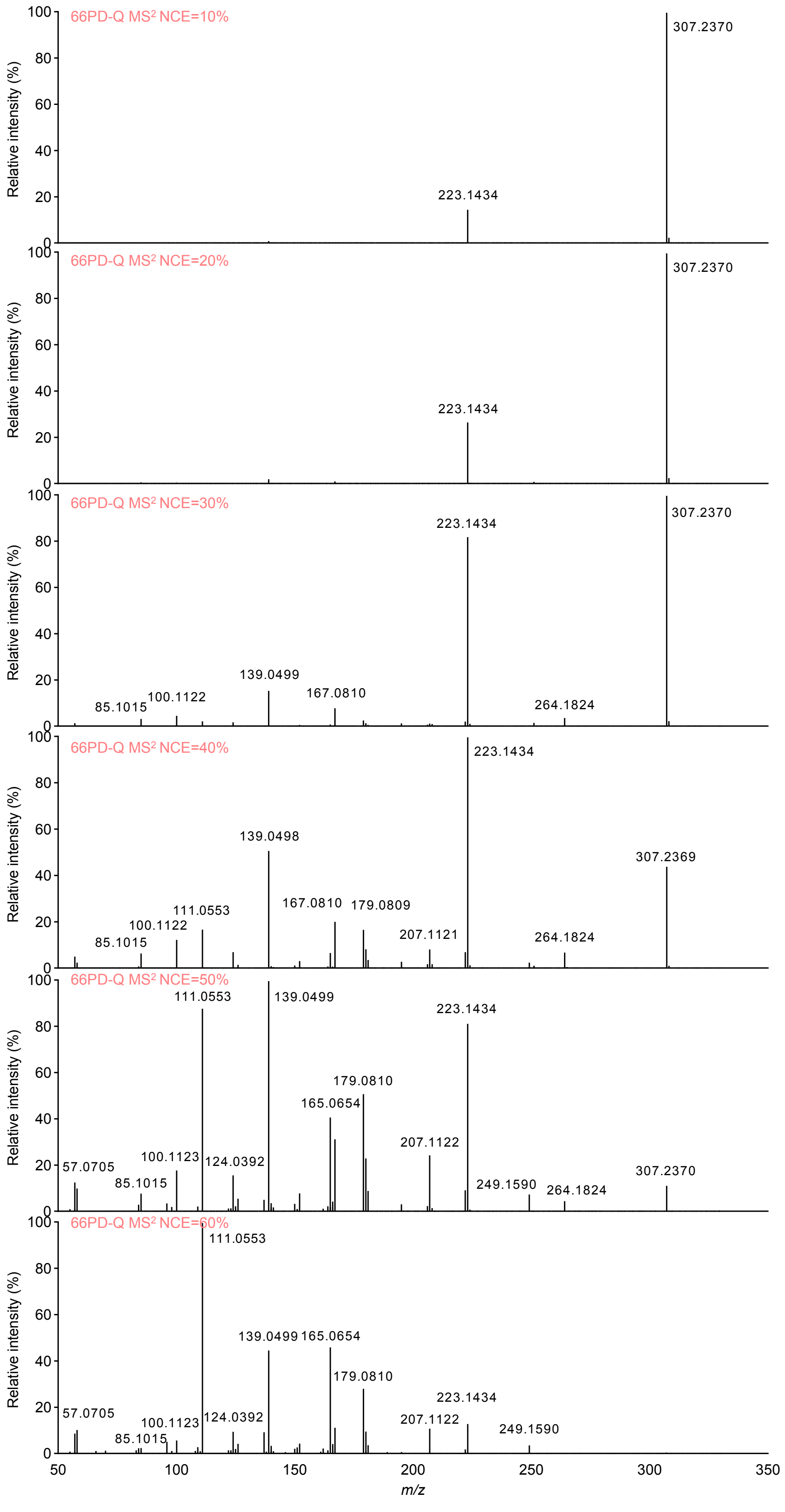

Figure S12. MS^2 spectra of 66PD-Q with normalized HCD collision energy (NCE) of 10-60%

References

1. Cao, G.; Wang, W.; Zhang, J.; Wu, P.; Zhao, X.; Yang, Z.; Hu, D.; Cai, Z., New Evidence of Rubber-Derived Quinones in Water, Air, and Soil. *Environ Sci Technol* **2022,** *56*, (7), 4142-4150.
2. Wang, W.; Cao, G.; Zhang, J.; Wu, P.; Chen, Y.; Chen, Z.; Qi, Z.; Li, R.; Dong, C.; Cai, Z., Beyond Substituted p-Phenylenediamine Antioxidants: Prevalence of Their Quinone Derivatives in PM2.5. *Environ. Sci. Technol.* **2022,** *56*, (15), 10629-10637.
3. U.S. EPA., EPI Suite™-Estimation Program Interface (Version 4.11). Available at: https://www.epa.gov/tsca-screening-tools/epi-suitetm-estimation-program-interface (*accessed* August 23, 2021)
4. Tian, Z.; Zhao, H.; Peter, K. T.; Gonzalez, M.; Wetzel, J.; Wu, C.; Hu, X.; Prat, J.; Mudrock, E.; Hettinger, R.; Cortina, A. E.; Biswas, R. G.; Kock, F. V. C.; Soong, R.; Jenne, A.; Du, B.; Hou, F.; He, H.; Lundeen, R.; Gilbreath, A.; Sutton, R.; Scholz, N. L.; Davis, J. W.; Dodd, M. C.; Simpson, A.; McIntyre, J. K.; Kolodziej, E. P., A ubiquitous tire rubber-derived chemical induces acute mortality in coho salmon. *Science* **2021,** *371*, (6525), 185-189.
5. Zhao, H. N.; Hu, X.; Gonzalez, M.; Rideout, C. A.; Hobby, G. C.; Fisher, M. F.; McCormick, C. J.; Dodd, M. C.; Kim, K. E.; Tian, Z.; Kolodziej, E. P., Screening p-Phenylenediamine Antioxidants, Their Transformation Products, and Industrial Chemical Additives in Crumb Rubber and Elastomeric Consumer Products. *Environ Sci Technol* **2023,** *57*, (7), 2779-2791.
6. Hughey, C. A.; Hendrickson, C. L.; Rodgers, R. P.; Marshall, A. G.; Qian, K., Kendrick mass defect spectrum: a compact visual analysis for ultrahigh-resolution broadband mass spectra. *Anal Chem* **2001,** *73*, (19), 4676-81.
7. Wang, W.; Zhang, Y.; Jiang, B.; Chen, Y.; Song, Y.; Tang, Y.; Dong, C.; Cai, Z., Molecular characterization of organic aerosols in Taiyuan, China: Seasonal variation and source identification. *Sci. Total Environ.* **2021,** *800*, 149419.
8. Hu, X.; Zhao, H.; Tian, Z.; Peter, K. T.; Dodd, M. C.; Kolodziej, E. P., Chemical characteristics, leaching, and stability of the ubiquitous tire rubber-derived toxicant 6PPD-quinone. *Environ. Sci. Processes Impacts* **2023,** *25*, (5), 901-911.
